# Stereotactic ablative radiotherapy-driven immunosuppression is associated with poorer progression-free survival in cancer patients

**DOI:** 10.1007/s00262-025-04218-6

**Published:** 2025-12-18

**Authors:** Jessica Oliver, Hannah Reed, Lorenzo Capitani, Ashley Poon-King, Ashleigh Young, Stefan Milutinovic, Mateusz Kuczynski, Owen Nicholas, Thomas Rackley, Catherine Pembroke, Andrew Godkin, Awen M. Gallimore

**Affiliations:** 1 Systems Immunity University Research Institute, Cardiff Cancer Research Partnership, Cardiff, UK; 2 Velindre Cancer Hospital, Cardiff, UK; 3 Swansea Bay University Health Board, Swansea, UK; 4 ImmunoServ Ltd, Cardiff Medicentre, Cardiff, UK

**Keywords:** Radiotherapy, Stereotactic ablative radiotherapy, Oligometastatic disease, T cells, Neutrophil-to-lymphocyte ratio, Antigen-specific response

## Abstract

**Background:**

The landscape of cancer treatment has evolved rapidly within the last 50 years, and whilst radiotherapy, chemotherapy, and surgery remain the mainstay treatment options, there has been a shift towards using immunotherapy alone or in combination with other treatment modalities. There is an emerging paradigm that radiotherapy is immunogenic, driving stimulation of antigen-specific T cells capable of recognising tumour cells at distal sites to the treatment location.

**Methods:**

Whole blood samples were collected from patients with primary and oligometastatic cancer before, during, and after treatment with stereotactic ablative radiotherapy (SABR). Using clinical full blood counts, multiparameter flow cytometry, Luminex, and ELISpot assays, this study explored the impact of SABR on systemic immune cell composition, inflammatory markers, and antigen-specific T cell responses.

**Results:**

We identified striking systemic changes collectively indicating profound SABR-driven immunosuppression. Such changes were characterised by pronounced and sustained lymphopenia which included loss of CD4^+^ and CD8^+^ T cells, B cells, and natural killer (NK) cells accompanied by an overall decline in effector T cell responses to common recall and cancer antigens. This loss of lymphocytes drove a rise in the neutrophil-to-lymphocyte ratio (NLR), which was associated with poorer progression-free survival (PFS) if increased from baseline. A higher dosage of radiation and treatment to a larger area were both associated with more pronounced lymphocyte loss, a concomitant NLR increase, and poorer PFS, particularly in individuals with liver lesions.

**Conclusions:**

These findings support a role for lymphocytes in preventing disease progression after SABR and suggest that a change to clinical practice to spare lymphocytes from the toxic effects of irradiation may have beneficial effects for patients.

**Supplementary Information:**

The online version contains supplementary material available at 10.1007/s00262-025-04218-6.

## Background

There has been a significant increase in cancer treatment options in recent years due to advancements in genetic profiling and immunotherapy [1–5]. Moreover, combining different treatment types such as surgery, chemotherapy, radiotherapy (RT), and immunotherapy for optimal results can improve treatment success [6–8]. Despite the increase in treatment options for cancer, metastatic disease is still a major challenge accounting for around 90% of cancer-related deaths [9]. Although survival rates in individuals with metastatic cancer are typically poor, there is a suggestion that so-called oligometastatic disease, defined as a clinically distinct state of disease characterised by limited spread of malignant cells to a confined number of locations, may be amenable to localised treatments in a way that widespread metastatic disease is not [10]. Treatments to these lesions can be curative [11–13], whereas widespread metastatic disease is typically seen as incurable, and treatment is usually administered palliatively, highlighting the clinical separation in these two disease states.

Many patients with oligometastatic disease are now being treated with stereotactic ablative radiotherapy (SABR) [14]. SABR delivers high doses of radiation to the tumour whilst minimising the dose to surrounding healthy tissue. The high precision afforded by SABR induces less damage to surrounding cells, and the high dose enables treatment to be completed in fewer sessions than conventional RT [15–17]. SABR was adopted into the clinic after multiple studies demonstrated high rates of local control, improved progression-free survival (PFS), and relatively low toxicity [18]. The SABR-COMET trial was a phase II study conducted across multiple institutions worldwide that enrolled 99 patients with oligometastatic cancer [19]. PFS in the SABR arm was double that of the standard of care (SoC) arm (12 months vs 6 months), and overall survival (OS) was over a year longer (41 vs 28 months) [20]. Despite the improved clinical outcome observed in this study, the impact of ablative strategies on disease state and patient survival remains unclear due to heterogeneous outcomes [21].

Accumulating evidence suggests that RT, including SABR, may stimulate cancer antigen-specific T cells capable of recognising and targeting tumours that are not in the vicinity of the localised treatment (termed the abscopal effect) [22]. This emerging paradigm has fuelled research into approaches that enhance the immunogenic potential of RT to improve patient outcomes [23–25]. At present, we are limited in our ability to successfully progress these approaches due to a lack of understanding of how SABR affects the immune system, particularly the behaviour of antigen-specific T cells. To address this, we conducted a longitudinal study to assess the impact of SABR on the immune system in patients treated for primary or oligometastatic disease. Blood samples were collected before, during, and after SABR treatment and analysed for immune cell numbers and function. The study enabled us to identify SABR-driven perturbations to innate and adaptive immune cell populations and to assess these changes in relation to tumour recurrence and PFS.

## Methods

### Recruitment of patients to the SABR_IT study

Ethical approval for the SABR_IT study was obtained from the Integrated Research Application System (IRAS) in January 2021 (project ID 280149). Patients above 18 years old with a primary or oligometastatic cancer diagnosis that were eligible for ablative RT at Velindre Cancer Centre (VCC), Cardiff, Wales, were identified at multidisciplinary team (MDT) meetings. These patients were given a patient information sheet and, upon providing consent, were enrolled into the study. Patients under 18 years old or with a severe immune deficiency e.g. AIDs, anti-rejection transplant drugs, or high-dose corticosteroids, were not eligible for the study. Tumour biopsies were not part of the treatment regime for this cohort of patients, and thus, biopsies were not collected for this study. Instead, whole blood samples were collected before, during, and after treatment to investigate the systemic immune effects of SABR. 33 patients were recruited, with 2 unable to continue in the study after enrolment due to progression of disease prior to the commencement of SABR. As such, 31 patients enrolled on the study were evaluable; patient characteristics are shown in Table 1. Further details of all measured patient characteristics stratified by progression status are shown in Supp. Tables 1–3. Any systemic anti-cancer therapy received before, during, or after the study is highlighted in Supp. Table 4.

**Table 1:** Characteristics of Patients Recruited to the SABR_IT Study

**Age (Years)**	
Median age (range)	69 (49–85)
**Sex**	
Male	26 (83.9%)
Female	5 (16.1%)
**WHO Performance Status**	
0	12 (38.7%)
1	15 (48.4%)
2	4 (12.9%)
**Primary Cancer Site**	
Liver	9 (29.0%)
Colorectal	7 (22.6%)
Prostate	8 (25.8%)
Bladder	2 (6.5%)
Kidney	2 (6.5%)
Lung	1 (3.2%)
Breast	1 (3.2%)
Skin	1 (3.2%)
**Site of Irradiation**	
Bone	8 (25.8%)
Lymph Node	7 (22.6%)
Liver	12 (38.7%)
Lung	3 (9.7%)
**Treatment Indication**	
Metachronous OMD	17 (54.8%)
Synchronous OMD	1 (3.2%)
Oligoprogression	4 (12.9%)
Hepatocellular Carcinoma	9 (29.0%)
**Number of Irradiation Sites**	
1	27 (87.1%)
2	4 (12.9%)
**Gross Tumour Volume (Biggest)**	
Median (range)	5.9 ml (0.2 – 104 ml)
< 5 ml	12 (38.7%)
5 – 9.9 ml	7 (22.6%)
10 – 14.9 ml	5 (16.1%)
15 – 19.9 ml	2 (6.5%)
≥ 20 ml	5 (16.1%)
**Planning Target Volume (Biggest)**	
Median (range)	26.7 cc (7.4 – 241.8 cc)
< 20 cc	9 (29.0%)
20 – 49 cc	12 (38.7%)
50 – 99 cc	5 (16.1%)
≥ 100 cc	5 (16.1%)
**Biological Effective Dose**	
Median (range)	60 Gy (43.2 – 151.2 Gy)
< 60 Gy	5 (16.1%)
60–79 Gy	11 (35.5%)
80–99 Gy	6 (19.4%)
100–119 Gy	8 (25.4%)
≥ 120 Gy	1 (3.2%)
**Dose and Fractionation**	
24–30 Gy in 3 #	14 (45.2%)
24–30 Gy in 5 #	2 (6.5%)
31–50 Gy in 3 #	1 (3.2%)
31–50 Gy in 5 #	11 (35.5%)
51–60 Gy in 3 #	1 (3.2%)
51–60 Gy in 5 #	1 (3.2%)
51–60 Gy in 8 #	1 (3.2%)
**Systemic Treatment**	
Yes	5 (16.1%)
No	26 (83.9%)

Individual characteristics (n = 31) shown as raw numbers with the percentage of the cohort in brackets. OMD = oligometastatic disease, Gy = Gray, # = fractions, biggest refers to the metastatic lesion with the largest volume if the individual has more than 1 lesion being treated with SABR

### Blood sample collection and cell counts

30–50 ml of blood was collected in sodium heparin blood collection tubes (Becton Dickinson Cat#368480) at the participants’ initial planning scan (time point (TP) 1), immediately before the first fraction of SABR (TP2), just before their final fraction of SABR (TP3), and 4–6 weeks post-treatment (TP4). At each time point, an additional sample of blood was taken, and a full blood count (FBC) was performed by the clinical laboratory at VCC. A whole blood count (WBC) was also performed using the human TBNK 6-colour cocktail antibody (BioLegend, Cat#391503) according to the manufacturer’s instructions to measure proportions of lymphocytes (CD4^+^ and CD8^+^ T cells, B cells, and NK cells), monocytes, and granulocytes in whole blood (Supp. Fig. 1A). Data acquired from pre-SABR samples (TP1 and TP2) were combined to give an average ‘before’ data point for each patient and compared to TP3 (during) and TP4 (after). 1 patient was not available for a TP4 sample and was consequently excluded from the analysis.

### Preparation of peripheral blood mononuclear cells

Peripheral blood mononuclear cells (PBMCs) were isolated from whole blood by density gradient centrifugation using Lymphoprep (STEMCELL Technologies, Cat#07851). Plasma was collected from each sample and stored in liquid nitrogen for future analysis. PBMCs were collected, and any remaining red blood cells were lysed (BioLegend Cat#420301). Purified viable PBMCs that had been filtered through a 70 µM filter were counted by addition of propidium iodide (PI) (Sigma-Aldrich, Cat#P4864) and acquired on the NovoCyte Autosampler (Agilent Technologies) to provide an absolute cell count per μl.

### IFN-γ enzyme-linked ImmunoSpot (ELISpot) assays

ELISpot assays provide a robust measurement of cytokine-secreting cells at the single-cell level. A coloured spot on the membrane of the well is indicative of a single cytokine-secreting cell directly from an in vivo setting. For this reason, ELISpots are a sensitive assay for exploring antigen-specific T cells responses [26,27]. IFN-γ was measured as it is a cytokine that is released upon antigen-specific activation of T cells. ELISpot assays were performed with the Mabtech IFN-γ ELISpot kit (Mabtech Cat#3420-2A) using a 96-well filter plate with a 0.45 µm pore hydrophobic PVDF membrane (Millipore Cat#MAIPS4510). Briefly, wells were pre-coated with anti-IFN-γ antibodies, and for an ex vivo assay, 2.5 × 10^5^ PBMCs were added to each well and stimulated with peptide at a concentration of 1–10 µg / peptide / ml for 16 hours in duplicate. Cells and peptide were removed, and the wells were incubated with detection and streptavidin-conjugated antibodies before the addition of a substrate (Mabtech, Cat#3650–10) to visualise spots formed by IFN-γ production. Once wells were dry, spots were enumerated with the CTL ImmunoSpot SC Suite software on the ImmunoSpot S6 analyser (Cellular Technology Limited). For a cultured ELISpot, 2 × 10^5^ PBMCs were added to the wells of a round-bottomed 96-well cell culture plate in triplicate (Thermo Scientific Cat#163320) and stimulator peptides were added at 1–10 µg / peptide / ml and cultured for 10–14 days, with CTL media supplemented with 36 IU of IL-2 replenished every 3 days. An ELISpot assay was performed as described above by pooling cells from triplicate wells and using 25,000 cultured cells per well.

For ex vivo ELISpots, a positive response was defined as at least 10 spot-forming cells (SFC) per 1 × 10^6^ cells after the subtraction of the background (SFC in the negative control well) and at least 1.5 times the number of background spots. For cultured ELISpots, a positive response was defined as at least 20 SFC per 1 × 10^5^ cells following subtraction of the background of that condition (number of SFC from the cells that were not re-stimulated) and at least 2 times the number of background spots. The quality controlled raw values were exported into an excel file where the number of positive and negative responses were calculated. These values were inputted into Graphpad Prism 10.2.2 for graphical analysis. 3 patients were excluded from the analysis as measurements were not taken at all time points.

### Peptide pools

PBMCs were stimulated with HLA-agnostic peptide pools. The ELISpot plate contained a negative control (no peptide added) and phytohemagglutinin (PHA) added as a positive control (Roche, Cat#11249738001). Peptide pools of common recall viral antigens were used as additional positive controls and a marker of T cell function [28,29] as most individuals are able to mount a measurable response against them [30]. The CEF peptide pool (Mabtech, Cat#3616-1) which contains 23 HLA-I restricted epitopes from **C**ytomegalovirus, **E**pstein–Barr virus, and in**f**luenza virus was used to measure CD8^+^ T cell responses. The CEFTA peptide pool (Mabtech, Cat#3617-1) contains 35 HLA-II restricted peptides from human **C**ytomegalovirus, **E**pstein–Barr virus, in**f**luenza virus, **T**etanus toxin, and **A**denovirus 5 and stimulates CD4^+^ T cell responses). The lyophilised peptide pool was dissolved in dimethyl sulfoxide (DMSO) and further diluted in sterile phosphate-buffered saline (PBS) to give 200 μg / ml of each peptide. Peptide pools for trophoblast glycoprotein (5T4), DnaJ heat-shock protein family member 7 (DNAJB7), Carcinoembryonic Antigen-Related Cell Adhesion Molecule 3 (CEACAM3), and Zinc Finger SWIM-Type Containing 1 (ZSWIM1) (GLBiochem, China) consisted of 20-amino-acid-long peptides that overlapped by 10 amino acids to cover the entire protein (Supp. Table 5).

### Flow cytometry

PBMCs were stained with a panel of antibodies (Supp. Table 6) as described previously [31]. Briefly, cells were stained with Live/Dead Fixable Aqua Dead Cell Stain (Invitrogen, Cat#L34957) and subsequently incubated with 2% normal rat serum as a blocking solution and antibody cocktails before being washed, fixed, and permeabilised (Fixation/Permeabilisation Buffer, Thermo Fisher Cat#00–5523-00). After being washed in permeabilisation buffer (Thermo Fisher Cat#00-5523-00) and resuspended in 2% normal rat serum, cells were stained with intracellular antibodies. Finally, after washing, cells were resuspended in FACS buffer and acquired on the Novocyte 3000 (Agilent Technologies).

### Flow cytometry data analysis

.fcs files were exported from NovoExpress and loaded into FlowJo 10.9.0 (Becton Dickinson) for gating analysis (Supp. Fig. 2). The table editor function was used to extract proportions of populations from parent and grandparent gates, and these values were used to analyse the data graphically in Graphpad Prism 10.2.2.

### Luminex multiplex assays

Multiplex assays were used to measure analyte concentrations in plasma samples from patients before, during, and after SABR. A list of the panels used is summarised in Supp. Table 7. Assays were conducted according to the ThermoFisher Procartaplex user guide. Plates were read with a Luminex200 with settings as per the user guide. The Bio-Plex Manager software was used to generate the standard curves and quantify the concentration of each analyte in each sample. In the case that the level of the analyte in the plasma was below that of the lower limit of quantification (LLOQ) on the standard curve, the software assigned the observed concentration as < OOR (out of range) and these values were set to 1 pg / ml. All data acquired on the Luminex200 was exported as an.xlsx file. Data for all analytes was collated into one file and saved as a .csv and uploaded into RStudio where all data was analysed and made into graphical format. Additional figures were produced in GraphPad Prism.

### Correlation matrices

A multivariate analysis was conducted to identify any associations between patient characteristics, immune cell populations, and T cell function. Correlation matrices were generated using Spearman’s rank correlation with Holm’s post hoc correction in R studio (Version 2024.09.0 + 375) to investigate these associations before, during, and after SABR.

## Results

### Overall survival and progression-free survival in SABR-treated patients

**Fig. 1:**
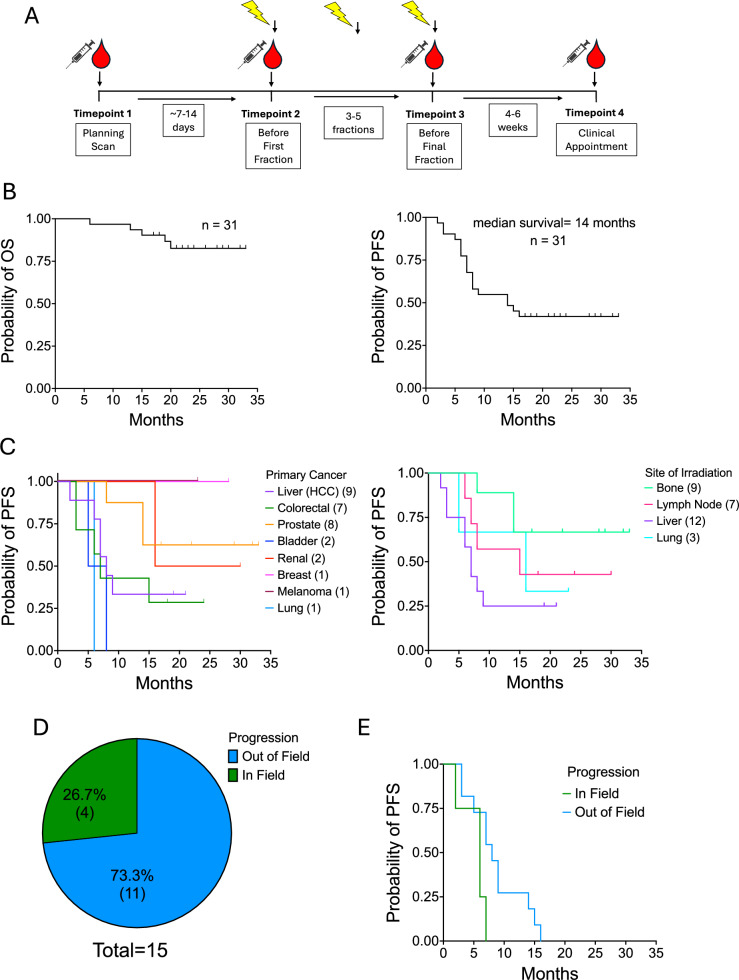
**Overall Survival and Progression-Free Survival in SABR-Treated Patients.** **A** Schematic depicting the timeline of the sample collection in the SABR_IT study, **B** Kaplan–Meier curves depicting the overall survival (OS) (left) and the progression-free survival (PFS) (right) of the SABR_IT patient cohort. **C** the PFS of the donors by primary cancer type (left) and the site of irradiation (right) with SABR (number of patients in brackets), **D** a pie chart depicting the proportion of patients (n = 15) that progressed in or out-of-field, **E** a Kaplan–Meier curve depicting the PFS in individuals who progressed in- or out-of-field (log-rank test), n = 31, ticks show censor

Blood was drawn from patients (n = 31) prior to commencement, during, and following completion of SABR (Fig. 1A) and were subsequently followed up for a maximum of 33 months, with a median follow-up period of 23 months. 5 patients passed away within the study period and 17 patients progressed (Fig. 1B–C). 8 primary cancer types were included in this study with breast, melanoma, and lung cancer represented by a single patient; neither the breast cancer patient nor the melanoma patient progressed within the study timeframe (Fig. 1C). Whilst most cancer progression occurred outside the field of treatment (Fig. 1D), PFS of in-field progressors was shorter (Fig. 1E). On the whole, PFS was shortest in individuals who received SABR to the liver for either primary liver cancer or liver metastases (Fig. 1C).

### Peripheral lymphocytes are diminished by SABR

**Fig. 2:**
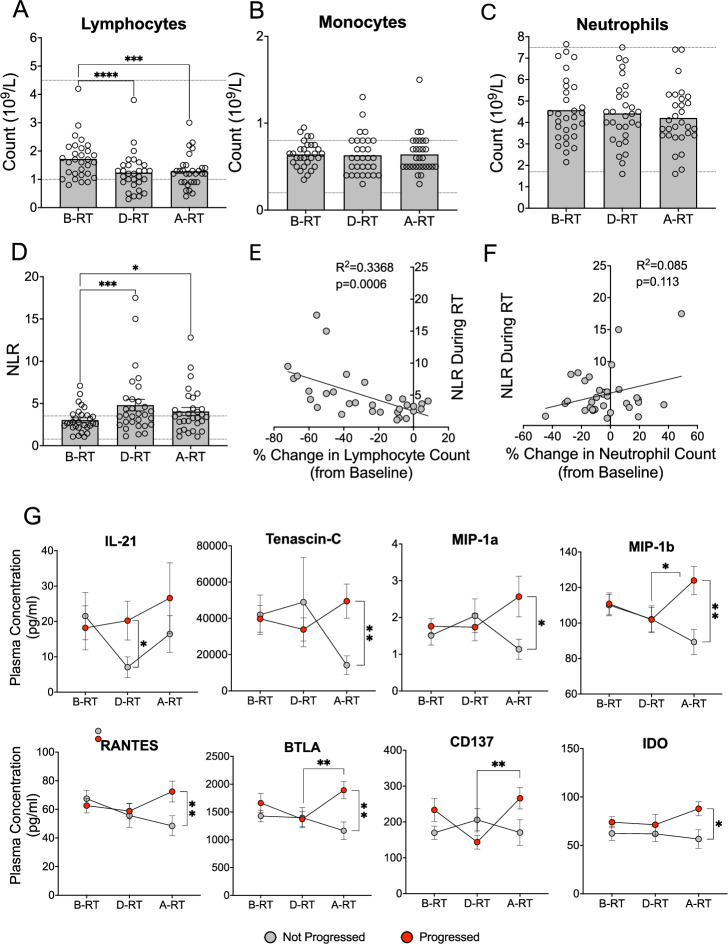
**Clinical Full Blood Counts and Whole Blood Counts Across SABR Treatment.** A clinical full blood count was conducted on whole blood samples to give a cell count expressed as × 10^9^ cells per litre for **A** lymphocytes, **B** monocytes, and **C** neutrophils before (B-RT), during (D-RT), and after (A-RT) radiotherapy (n = 30), Friedman tests and Dunn’s multiple comparison post hoc tests were performed, **D** the neutrophil-to-lymphocyte ratio (NLR) across SABR treatment (n = 30), a Friedman test with a Dunn’s multiple comparison post hoc test was performed, linear regression analyses depicting **E** the percentage change in lymphocyte count from baseline vs the NLR during SABR treatment and **F** the percentage change in neutrophil count from baseline vs the NLR during SABR treatment in the SABR_IT patient cohort, **G** plasma analyte concentrations in progressors (red) and non-progressors (grey) before, during, and after SABR. Bars and dots show mean value, error bars depict SEM, dotted lines indicate normal ranges, * = *p* < 0.05, ** = *p* < 0.01, *** = *p* < 0.001, **** = *p < 0.0001*

A FBC was performed with each blood sample collection appointment by the hospital clinical laboratory which provided details of lymphocyte, monocyte, and neutrophil counts at two baseline points (TP1 and TP2) and in further samples taken during (TP3) and after (TP4) the SABR treatment (Fig. 2A–C). Little variation was observed in counts across pre-treatment samples, and as such, values were combined to give a ‘before RT’ measurement that was compared to TP3 (during RT) and TP4 (after RT). Overall, we observed that SABR led to a marked reduction in lymphocyte counts across all patients (*p* = <0.0001), with many experiencing levels that fell below the lymphopenia threshold (< 1 × 10^9^ cells / L) for up to 4–6 weeks following the completion of treatment *(p* = 0.0002) (Fig. 2A). A drop in counts was observed within T cell, B cell, and NK cell populations (Supp. Fig. 1B). Conversely, monocyte and neutrophil counts remained relatively static and stayed within normal ranges (Fig. 2B–C. Among the T cells remaining after SABR, there was a shift in the subset proportions with a slight increase of CD4^+^ T cells and a decrease in CD8^+^ T cells (Supp Fig. 3A–C). We investigated whether SABR resulted in any changes to PD-1 expression on T cell subsets as a potential marker of immune activation or suppression. However, no significant changes were observed (Supp. Fig. 3D–F) consistent with the findings of Geboers and colleagues [32].

Since NLR is considered a measure of inflammation and has been linked to pathology and disease progression in both inflammatory conditions and cancer, a longitudinal analysis of NLR was conducted. Consistent with diminished lymphocyte counts, a SABR-driven rise in NLR was observed in many patients (*p* = 0.0001), with ratios surpassing reported normal ranges (0.78–3.53) [33] and which were sustained for at least 4–6 weeks after treatment finished (*p* = 0.0135) (Fig. 2D). The rise in NLR was clearly due to the drop in lymphocyte numbers given the significant negative correlation (*p* = 0.0006) between the percentage change from baseline in lymphocyte count, but not neutrophil count, and NLR during SABR (Fig. 2E–F). Moreover, Luminex multiplex assays were performed to measure the concentrations of 55 proteins in the plasma of patients collected before, during, and after SABR (Supp. Table 7). No significant changes in inflammatory markers were observed in the plasma of patients during SABR (Supp Fig. 4). Whilst pre-treatment levels of these analytes were comparable, significant differences between progressors and non-progressors did emerge for 8 of the measured analytes following SABR (Fig. 2G). Hierarchical clustering, however, revealed no unique SABR-driven cytokine signature which could distinguish progressors from non-progressors (Supp. Fig. 5A–B).

Whilst the patient group comprised several different tumour types as well as different sites of irradiation, it was nevertheless possible to observe that a significant SABR-driven NLR was more likely in HCC patients and in those receiving SABR for liver metastases (Supp. Fig. 6A–D). Those who received SABR to bone metastases did not experience a marked loss of lymphocytes. Most of these patients had primary prostate cancer, which is reflected in their stable lymphocyte count and NLR. Moreover, SABR had minimal impact on the NLR of patients with colorectal cancer (CRC) or receiving treatment to a lymph node. The number of patients with other cancer types was too small to draw conclusions.

### A SABR-driven raise in NLR is associated with poorer progression-free survival

**Fig. 3:**
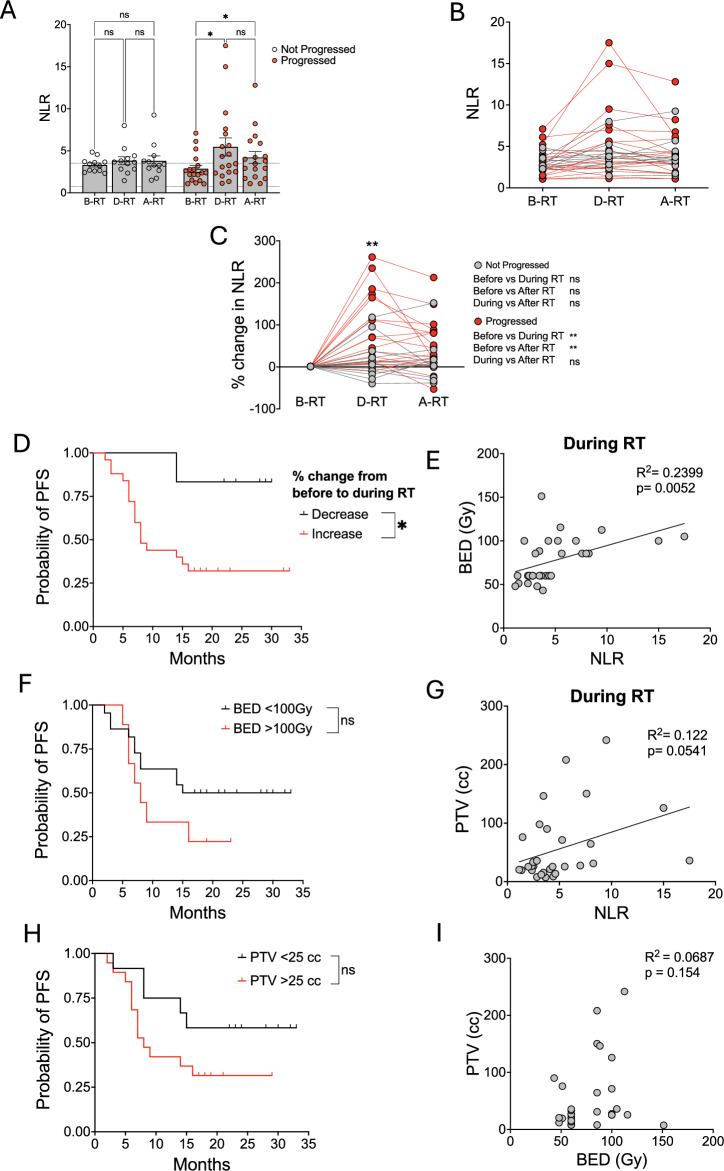
**A SABR-Driven Raise in NLR is Associated with Poorer Progression-free Survival.** **A** Neutrophil-to-lymphocyte ratio (NLR) stratified by progression status before (B-RT), during (D-RT), and after (A-RT) SABR, a two-way ANOVA with Tukey’s multiple comparison post hoc test was performed, **B** the change in NLR of individual patients before, during, and after SABR with patients who progressed (red) and who did not (grey) and **C** the percentage change in NLR from baseline of each individual patient with patients who progressed (red) and those who did not (grey), two-way ANOVAs with Tukey’s multiple comparison post hoc test were performed, **D** A Kaplan–Meier curve depicting the progression-free survival (PFS) of those who had a percentage decrease in NLR from baseline to during their treatment (decrease—black) and those that had a percentage increase in NLR from baseline to during their treatment (increase—red), **E** a linear regression depicting the relationship between biological effective dose (BED) and NLR during SABR, **F** a Kaplan–Meier curve depicting the PFS of those who had a BED < 100 Gy (black) and > 100 Gy (red), **G** a linear regression depicting the relationship between planning target volume (PTV) and NLR during SABR, **H** a Kaplan–Meier curve depicting the PFS of those who had a PTV < 25 cc (black) and > 25 cc (red), **I** a linear regression depicting the relationship between PTV and BED, n = 30, bars show mean value, error bars depict standard error of the mean (SEM), dotted lines depict normal ranges, log-rank (Mantel–Cox) tests were performed on all Kaplan–Meier curves * = *p* < 0.05, ** =  < 0.01, ns = not significant

Patients were classified as a progressor when scans demonstrated growth of an existing metastatic lesion and/or the emergence of a new deposit. Non-progressors comprised patients with disappearance or shrinkage of the irradiated lesion or who had stable disease. When NLRs were examined in progressors and non-progressors, we found that baseline NLR was no different between the groups (Fig. 3A–B). However, a significant increase in treatment-driven NLR (*p* = 0.0120), which was sustained following treatment cessation (*p* = 0.0208) was observed in progressors, whilst NLRs remained stable in non-progressors across treatment. Interestingly, in absolute terms, NLRs were not particularly higher in progressors compared to non-progressors (Fig. 3B), indicating that progression was not associated with NLR of itself, but rather with a significant SABR-driven percentage change in the ratio (Fig. 3C). We show that using an NLR cut-off of 4 was not predictive of treatment outcome at any time point (Supp Fig. 7A–C), but any percentage increase in NLR resulted in poorer PFS (*p* = 0.0328) (Fig. 3D). These findings indicate that a SABR-induced change in NLR, driven by a loss of lymphocytes, is indicative of poor prognosis; a pattern that was particularly pronounced in patients with liver tumours.

### The biological effective dose and planning target volume were associated with lymphocyte loss and poorer progression-free survival

We next examined the relationship between biological effective dose (BED) and planning target volume (PTV) with NLR and treatment outcome. Whilst BED considers fractionation schedule and total dose of the treatment, PTV defines the 3D area targeted for irradiation. Since an ablative dose of RT has been described previously as > 100 Gy [34,35], patients were grouped according to whether they received a BED ≥ 100 Gy or < 100 Gy, which varied across primary cancer type and treatment location (Supp. Fig. 8A–B). The median PTV within our patient group was 26.7 cubic centimetres (cc) and highest for the sole primary lung cancer patient and individuals receiving SABR to the liver (Supp. Fig. 9A–B). For our analysis, patients were divided into groups defined as either receiving a PTV of < or ≥ 25 cc.

A higher BED was associated with a higher NLR during treatment (*p* = 0.0052) (Fig. 3E). All patients that fell into the BED ≥ 100 Gy group exhibited a treatment-driven decrease in lymphocytes with a corresponding increase in NLR (Supp. Fig. 8C–D) and a trend for poorer PFS (Fig. 3F). In- or out-of-field progression was not influenced by BED (Supp. Fig. 8E). Patients with a higher PTV also exhibited a treatment-driven increase in NLR (as a result of lymphocyte loss) (Fig. 3G, Supp. Fig. 9C–D). Those receiving a PTV ≥ 25 cc had a median PFS of 8 months compared to an undefined PFS in the group with a PTV < 25 cc, however, this difference was also not significant (Fig. 3H). Moreover, a PTV of ≥ 25 cc was more associated with in-field progression compared to those that received < 25 cc, where no patients progressed in-field (Supp. Fig. 9E). Although PTV correlates with initial tumour size (Supp. Fig. 9F), there is no association between PTV and BED (Fig. 3I), showing that they impact NLR and progression independently. Although higher PTV and BED showed a trend towards worse PFS, neither were statistically significant, suggesting that the most predictive factor of progression was a change in NLR.

### SABR diminishes antigen-specific effector but not memory T cell responses

To determine whether SABR impacted the magnitude of T cell responses specific for cancer- and virus-specific antigens,  ex vivo ELISpot assays were conducted using PBMCs purified from blood samples before, during, and after treatment. To assess responses to cancer antigens, T cell responses to overlapping peptides covering tumour associated antigens (TAA) 5T4, DNAJB7, CEACAM, and ZSWIM1 were measured, whilst CEF and CEFTA peptide pools were used to measure T cell responses to common recall antigens.

**Fig. 4:**
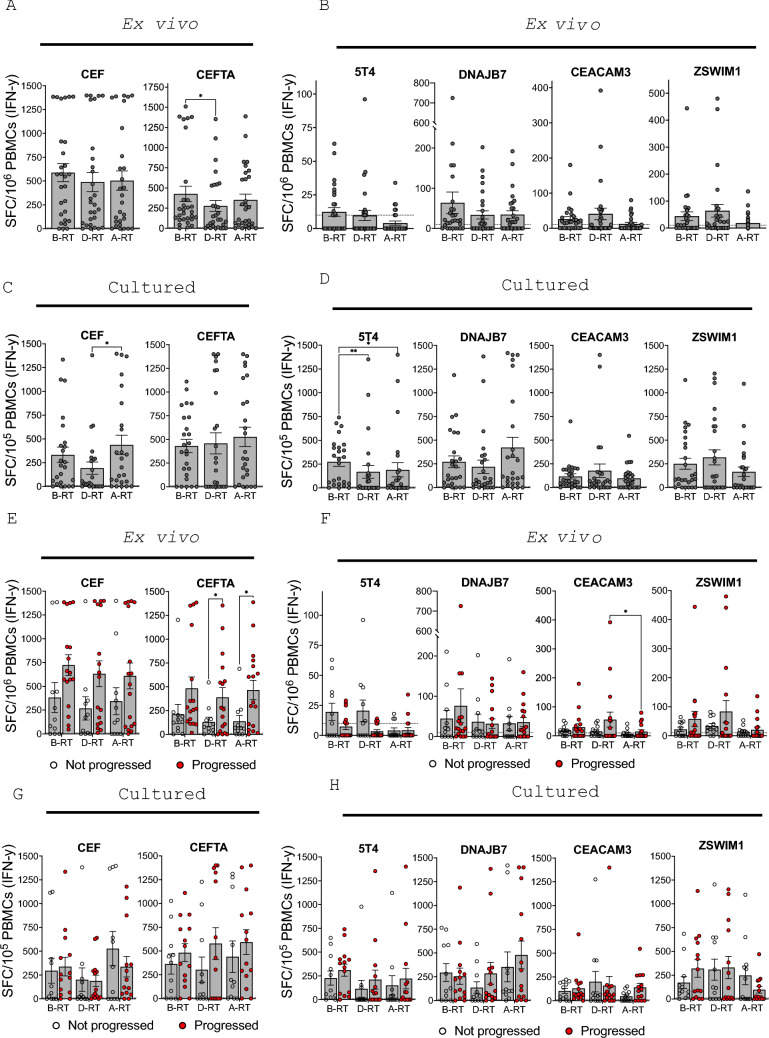
**IFN-γ Responses to Common Viral Antigens and Tumour Associated Antigens.** IFN-γ spot-forming cells (SFC) per 10^6^ PBMC in response to ex vivo stimulation with **A** the CEF and CEFTA peptide pools, **B** the 5T4, DNAJB7, CEACAM3, or ZSWIM1 peptide pools, and the SFC per 10^5^ PBMC in response to re-stimulation following culture with **C** the CEF and CEFTA peptide pools, **D** the 5T4, DNAJB7, CEACAM3, or ZSWIM1 peptide pools. Data points show mean value before (B-RT), during (D-RT) and after (A-RT) radiotherapy, error bars depict standard error of the mean (SEM). Friedman tests with Dunn’s multiple comparisons were performed. IFN-γ SFC per 10^6^ PBMC in progressors (red) and non-progressors (clear circles) in response to ex vivo stimulation with and **E** CEF and CEFTA **F** 5T4, DNAJB7, CEACAM3, ZSWIM1 peptides pools, and IFN-γ spot SFC per 10^5^ PBMC in progressors and non-progressors in response to re-stimulation following culture with **G** CEF and CEFTA and (H) 5T4, DNAJB7, CEACAM3, ZSWIM1 peptides pools. Bars show mean value before, during, and after radiotherapy, error bars depict SEM, two-way ANOVAs with Tukey’s multiple comparison post hoc tests were performed. * = *p* < 0.05, ** = *p* < 0.01. Dashed line shows the positive response cut-off value

These experiments revealed that CD8^+^ T cell responses to the CEF peptide pool were unaffected by SABR whilst CD4^+^ T cell responses to HLA-II-restricted peptides in the CEFTA peptide pool were significantly reduced (*p* = 0.0213) (Fig. 4A). Ex vivo responses to 5T4 in cancer patients were low at baseline and were further reduced at 4–6 weeks after SABR (Fig. 4B). Whilst overall responses to DNAJB7, CEACAM3 and ZSWIM1 did not significantly change during SABR, IFN-γ production dropped below pre-treatment levels 4–6 weeks following the end of treatment (Fig. 4B). Overall, we found no evidence of SABR-driven immunogenicity, rather the results of the ELISpot assays indicated that effector T cell responses are generally diminished following SABR.

Next, central memory T cell responses to the same panel of common recall antigens and TAAs were measured using cultured ELISpots [36,37]. PBMCs were stimulated with peptide and cultured for 10–14 days to allow expansion of antigen-specific memory cells. ELISpot assays were subsequently performed in all but 6 donors who were excluded from analysis as measurements were not taken at all time points. CD8^+^ T cell responses to common recall antigens reduced slightly during SABR but significantly increased 4–6 weeks following treatment, whereas CD4^+^ T cell responses remained constant (Fig. 4C). Overall, robust memory T cell responses were observed to all TAAs, with the exception of 5T4, where a significant drop in responses was seen during (*p* = 0.0071) and after (*p* = 0.04) treatment (Fig. 4D). Cultured memory T cell responses to DNAJB7, CEACAM3, and ZSWIM1 remained unchanged.

Overall, our data indicate that effector T cells are more likely to be affected than central memory T cell responses as a result of SABR. Overall, there were no significant changes in responses across treatment in progressors or non-progressors for any of the antigens tested, other than a significant decrease in the ex vivo CEACAM3 responses after SABR in those who progressed (Fig. 4E-H).

Curiously, when measuring T cell responses to common recall antigens in progressors versus non-progressors, we found that the CD4^+^ T cell responses were significantly higher in progressors during (*p* = 0.0318) and after (*p* = 0.0106) treatment (Fig. 4E). There is evidence that infections such as Cytomegalovirus (CMV) can be reactivated during cancer [38] and that CMV reactivation can be a high-risk phenotype in cancer leading to disease progression [39]. In a separate study, Goerig and colleagues showed that in brain cancer, reactivation of CMV following chemoradiotherapy led to premature death [40]. It is therefore possible that the significantly higher levels of CD4^+^ T cells demonstrated in progressive patients may reflect virus reactivation. It has also been demonstrated that viral infections such as Epstein–Barr virus (EBV) can be reactivated with RT [41]. With these studies in mind, it is tempting to speculate that the significantly higher levels of CEFTA-specific CD4^+^ T cells demonstrated in progressive patients may reflect reactivation of viruses such as EBV and CMV. None of the patients in this study developed symptomatic viral infections, suggesting that although a decrease in ex vivo T cell responses to viral recall antigens was measured, this was not a clinically significant change.

### Multivariate analyses of patient characteristics, immune cell populations, and T cell function before, during, and after SABR

**Fig. 5:**
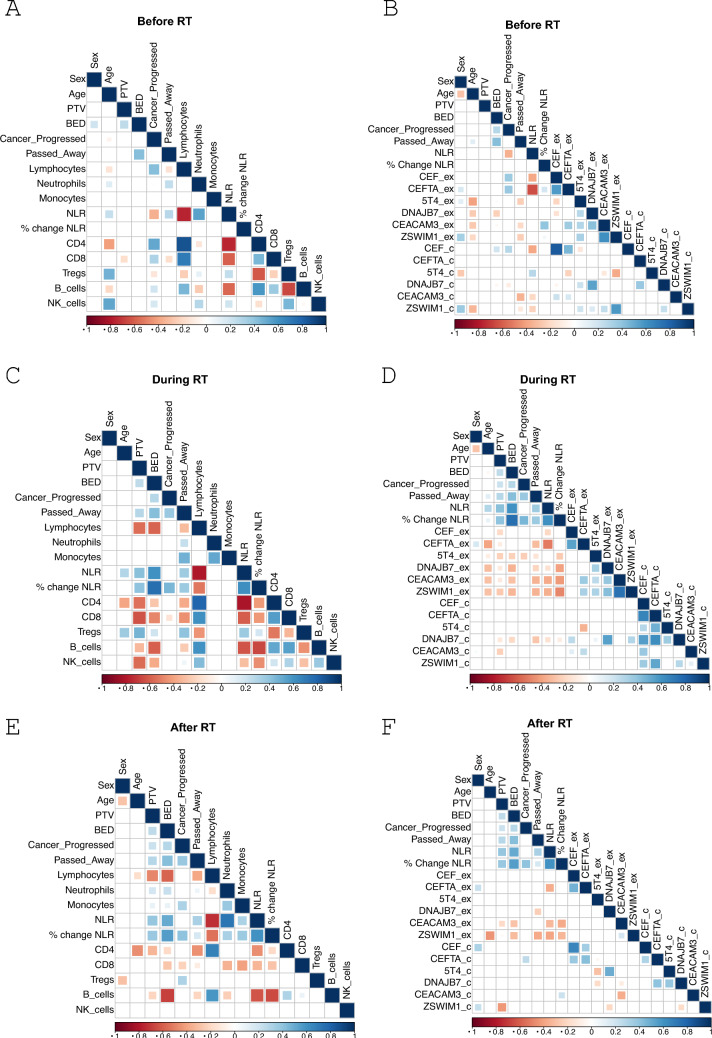
**Correlation Matrices to Show Associations Between Variables of the SABR_IT Study.** Spearman’s rank correlation was performed with Holm’s post hoc correction to identify positive (blue) and negative (red) correlations between patient characteristics, cell types from the clinical full blood counts and whole blood cell counts, and ex vivo and cultured ELISpot responses (**A**, **B**) before, (**C**, **D**) during, and (**E**, **F**) after SABR. (A bigger/darker square refers to a stronger correlation, ex = ex vivo ELISpot, c = cultured ELISpot, % change in NLR is the change from baseline neutrophil-to-lymphocyte ratio

Multivariate analyses performed on samples prior to SABR (baseline measurements) revealed correlations between NK and T_reg_ cell numbers and age, consistent with previous reports [42–44] (Fig. 5A). Furthermore, an inverse relationship was observed between baseline NLR and the number of T cells, particularly CD4^+^ T cells, which is further demonstrated by a negative relationship between NLR and ex vivo CEFTA-specific T cell responses (Fig. 5B). There was also a strong negative association between T_reg_ proportions and ex vivo 5T4 responses (Supp. Fig. 10). This is striking as our lab has previously shown that T cell responses to 5T4 are inhibited by T_reg_ cells in patients with CRC and that these responses are enhanced upon treatment of patients with low-dose cyclophosphamide which depletes T_regs_ [45–47].

Several trends emerged when analysing the data collected during SABR treatment. A higher BED and PTV were associated with poorer outcomes, and a decrease in lymphocytes leading to an increased percentage change in NLR was associated with disease progression (Fig. 5C). PTV was negatively correlated with all lymphocyte subsets, including CD4^+^ T cells, CD8^+^ T cells, B cells, and NK cells, except T_regs_ where the opposite was true. The analyses also showed that higher T_reg_ proportions correlated with lower ex vivo responses to cancer antigens (Supp. Fig. 11). BED correlated with a significant overall decrease in lymphocytes, but a higher number of T_regs_ (Fig. 5C) as well as lower ex vivo responses to TAAs (Fig. 5D) and poor OS. Similar correlations were observed when post-SABR measurements were analysed (Fig. 5E - F, Supp. Fig. 12). Overall, these results demonstrate that SABR does indeed drive significant changes to the immune system, and they appear to be primarily immunosuppressive. Moreover, these data clearly indicate that SABR-driven immunosuppression is linked to poor outcomes.

## Discussion

This study set out to identify signals of immune perturbation in cancer patients receiving SABR in order to improve our understanding of the effects of radiation on the immune system. The OS in this patient cohort was promising, with 84% of patients still alive by the end of the study, similar to the 3-year OS observed by Baker *et al*. in patients with oligometastatic cancer receiving SABR [48]. Moreover, at the 3-year follow-up point in the SABR_COMET trial, which evaluated the efficacy of SoC treatment versus SoC in addition to SABR, median OS had not yet been defined [20]. The median PFS in the SABR_IT study was 14 months, comparable to the median PFS of 11.4 months observed in the SABR arm of the SABR_COMET trial. Most incidences of tumour progression were outside of the treatment field as observed previously [49,50], however, 50% of progressive occurrences were in-field upon lymph node irradiation. Previous studies have highlighted that lymph node irradiation hinders the immune response and limits tumour control [51,52], and therefore, dosage and fractionation time should be carefully considered during planning to minimise this outcome.

In this study, we showed that a SABR-induced increase in NLR was a marker of poor outcome, supporting the conclusions of previous studies [53–55]. The SABR-driven rise in NLR was due to loss of lymphocytes rather than an increase in neutrophils, implying that the underpinning mechanism is linked to radiation-induced death of lymphocytes and not an inflammation-driven increase in neutrophils, a finding previously observed in lung cancer [56,57]. Supporting this interpretation of the data, longitudinal measurements of inflammatory markers in the plasma of patients before, during, and after SABR revealed no evidence of RT-driven inflammation. Lymphopenia post-SABR varied by radiation site, with rises in NLR predominantly seen in patients undergoing SABR to lung and liver and not to bone or lymph node, as previously observed [58].

Radiation-induced immune suppression (RIIS) has been described previously in multiple tumours sites, with both standard and hypofractionated RT schedules (e.g. SABR) [59–63]. Retrospective studies have shown that irradiating organs which contain pools of blood and therefore lymphocytes, such as the heart, lymph glands, and spine, typically result in higher rates of lymphopenia [64].

Several published studies have attempted to model the impact of organ-specific dosimetry on the immune system to develop methods of sparing immune cells [65,66]. Recent work has demonstrated that it is feasible to spare lymphocytes by optimising treatment plans to avoid blood and lymphocyte-rich organs for patients undergoing SABR [67–69]. Other strategies have been proposed to mitigate for lymphopenia during SABR, though none are currently in routine clinical practice. One approach might be the administration of cytokines e.g. IL-7, that may promote lymphocyte survival [70,71]. Furthermore, the immunosuppressive effects of high-dose radiation that have been previously reported [62,63] may be mitigated by giving low-dose radiation to reprogramme the tumour microenvironment (TME) to a more immune ‘hot’ phenotype and increase the susceptibility to immunotherapies [72–74]. Collectively, these findings are compelling, suggesting that a change in clinical practice to reduce lymphocyte loss would have beneficial effects for patients.

We observed that antigen-specific T cell responses decreased following SABR, a finding that was consistent with the results of Domouchtsidou *et al*. who noted that responses to mitogens and recall antigens were lowest 7 days after RT and remained below baseline levels a month later [75]. It has been previously reported that although all lymphocyte populations decrease upon irradiation, with a 50% lethal dose being just 2 Gy [76], naïve T cells appear to be more radio-sensitive than antigen-experienced and memory T cells [77–79]. Intratumoral T cells and tissue-resident memory T cells have also been shown to be more radio-resistant than circulating T cells [79] and can therefore continue to elicit tumour control. This study focused on peripheral lymphocyte populations and therefore cannot exclude the possibility that intratumoral T cells were not depleted in the same manner as the circulating lymphocytes. Nevertheless, the correlation matrices highlighted that ex vivo T cell responses were more highly impacted by BED and PTV compared to the cultured memory responses. Additionally, T_regs_ have also been shown to be more radio-resistant than other lymphocyte populations [77,80], perhaps explaining the increased proportion of T_regs_ observed following SABR and the decrease in ex vivo responses to common recall antigens and TAAs. These observations are limited to peripheral blood as due to the lack of biopsy material, RT-driven changes within the TME could not be investigated. Access to biopsies would allow exploration of the immunophenotype and TCR repertoires of tumour-infiltrating lymphocytes in response to RT, thereby providing further insight.

The reduction in the overall number of lymphocytes following SABR may hinder efficacy of anti-PD-1 therapies, as demonstrated by Kuge *et al. *who observed that baseline lymphocyte counts or recovery after chemo-RT was associated with improved PFS after administration of anti-PD-1 [81]. In addition to mitigating lymphocyte loss for potential increased efficacy of anti-PD-1 therapies, the proportions of T cells positive for other targetable cell surface markers such as CTLA-4 and LAG-3 should be investigated as an upregulation of an inhibitory marker following RT would provide a rationale for exploring immunotherapy interventions. Moreover, as proportions of T_regs_ were shown to increase with higher radiation doses, it would be worth exploring combinations with agents such as cyclophosphamide to deplete these populations [82] as done by Herrera and colleagues who have shown that low-dose RT in combination with low-dose cyclophosphamide and immunotherapy triggered T cell activation and infiltration in patients with immune-desert tumours [74].

Whilst the findings of this study are significant, there is heterogeneity in the cancer types included in this cohort, which is relatively small, and there may be additional baseline characteristics of the patients that we have not assessed that may be important for response to RT. Nevertheless, irrespective of previous treatments, there was no difference in lymphocyte counts or NLR prior to the commencement of SABR between those who did and did not go on to progress. We have demonstrated that despite heterogeneity in baseline characteristics, the percentage change in NLR during treatment remained a strong predictor of outcome. Furthermore, the definition of oligometastatic is poorly defined [83] and there is inclusion of patients with slow growing early tumours with a single metastatic lesion as well as others with a more aggressive cancer phenotype that has not responded to previous treatments. Future work is required to interrogate the individual patient treatment plans to assess organ-specific dosimetry to understand how this correlates with clinical outcomes. A larger, prospective study of a single cancer type or treatment site is now needed to determine the full prognostic impact of NLR. This information is essential for determining the need for mitigation strategies.

## Conclusions

Overall, we show that high-dose RT induces lymphocyte loss and an increase in NLR and a reduction in some antigen-specific T cell responses, highlighting areas that need further exploration for harnessing the RT response. Enhancing our knowledge in these areas of this research field will pave the way for enhanced treatment options that maximise the immune-mediated tumour rejection capabilities of an individual and improve clinical outcome and survival for patients with solid tumours.

## Supplementary Information

Below is the link to the electronic supplementary material.

Supplementary file1 (PPTX 3101 KB)

Supplementary file2 (PPTX 845 KB)

## Data Availability

The datasets used and/or analysed during the current study are available from 10.17035/cardiff.30510212.

## Abbreviations

5T4: Trophoblast glycoprotein
BED: Biological effective dose
CD: Cluster of differentiation
CEACAM3: Carcinoembryonic antigen-related cell adhesion molecule 3
CEF: Cytomegalovirus, epstein–barr virus, and influenza virus
CEFTA: Cytomegalovirus, epstein–barr virus, influenza virus, tetanus toxin, and adenovirus 5
CMV: Cytomegalovirus
CRC: Colorectal cancer
CTL: Cytotoxic T lymphocyte
DMSO: Dimethyl sulfoxide
DNAJB7: DnaJ heat-shock protein family member 7
EBV: Epstein–Barr virus
ELISpot: Enzyme-Linked ImmunoSpot
FBC: Full blood count
HCC: Hepatocellular carcinoma
IFN: Interferon
IL: Interleukin
IRAS: Integrated research application system
LLoQ: Lower limit of quantification
MDT: Multidisciplinary team
NK: Natural killer
NLR: Neutrophil-to-lymphocyte ratio
OOR: Out of range
OS: Overall survival
PBMC: Peripheral blood mononuclear cells
PFS: Progression-free survival
PI: Propidium iodide
PTV: Planning target volume
RIIS: Radiation-induced immune suppression
RT: Radiotherapy
SABR: Stereotactic ablative radiotherapy
SFC: Spot-forming cells
SoC: Standard of care
TAA: Tumour associated antigen
TME: Tumour microenvironment
TP: Time point
Treg: Regulatory T cell
VCC: Velindre Cancer Centre
WBC: Whole blood count
ZSWIM1: Zinc finger SWIM-type containing 1
