# Supplementary material for: Stereotactic ablative radiotherapy-driven immunosuppression is associated with poorer progression-free survival in cancer patients
Source: Cancer Immunol Immunother. 2025 Dec 18;75(1):3. doi: 10.1007/s00262-025-04218-6 (PMC12715060; doi:10.1007/s00262-025-04218-6)
Supplement: Supplementary file 1 — Supplementary file1 (PPTX 3101 KB) [file 262_2025_4218_MOESM1_ESM.pptx]

## Slide 1
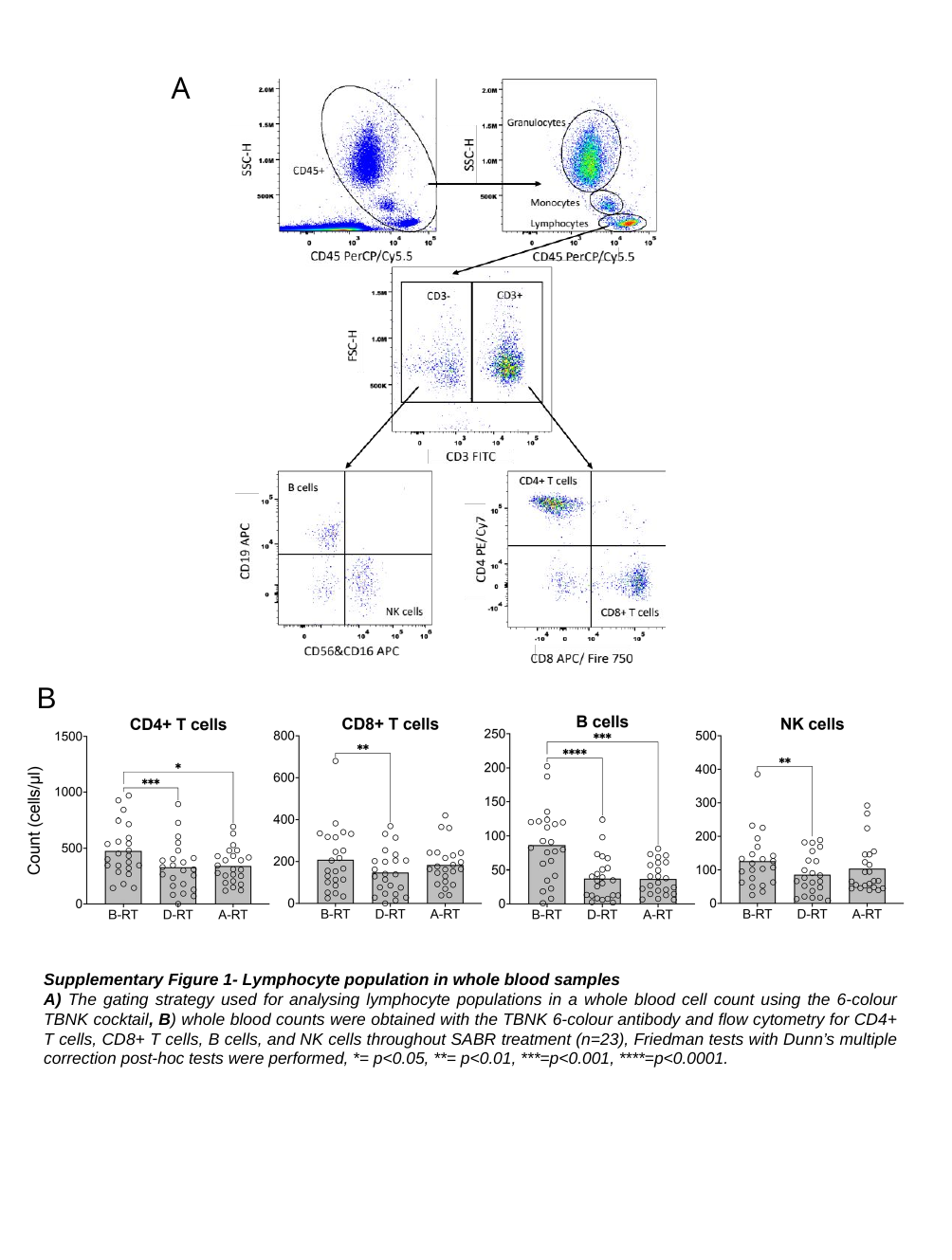

A
B
Supplementary Figure 1- Lymphocyte population in whole blood samples
A) The gating strategy used for analysing lymphocyte populations in a whole blood cell count using the 6-colour TBNK cocktail, B) whole blood counts were obtained with the TBNK 6-colour antibody and flow cytometry for CD4+ T cells, CD8+ T cells, B cells, and NK cells throughout SABR treatment (n=23), Friedman tests with Dunn’s multiple correction post-hoc tests were performed, *= p<0.05, **= p<0.01, ***=p<0.001, ****=p<0.0001.

## Slide 2
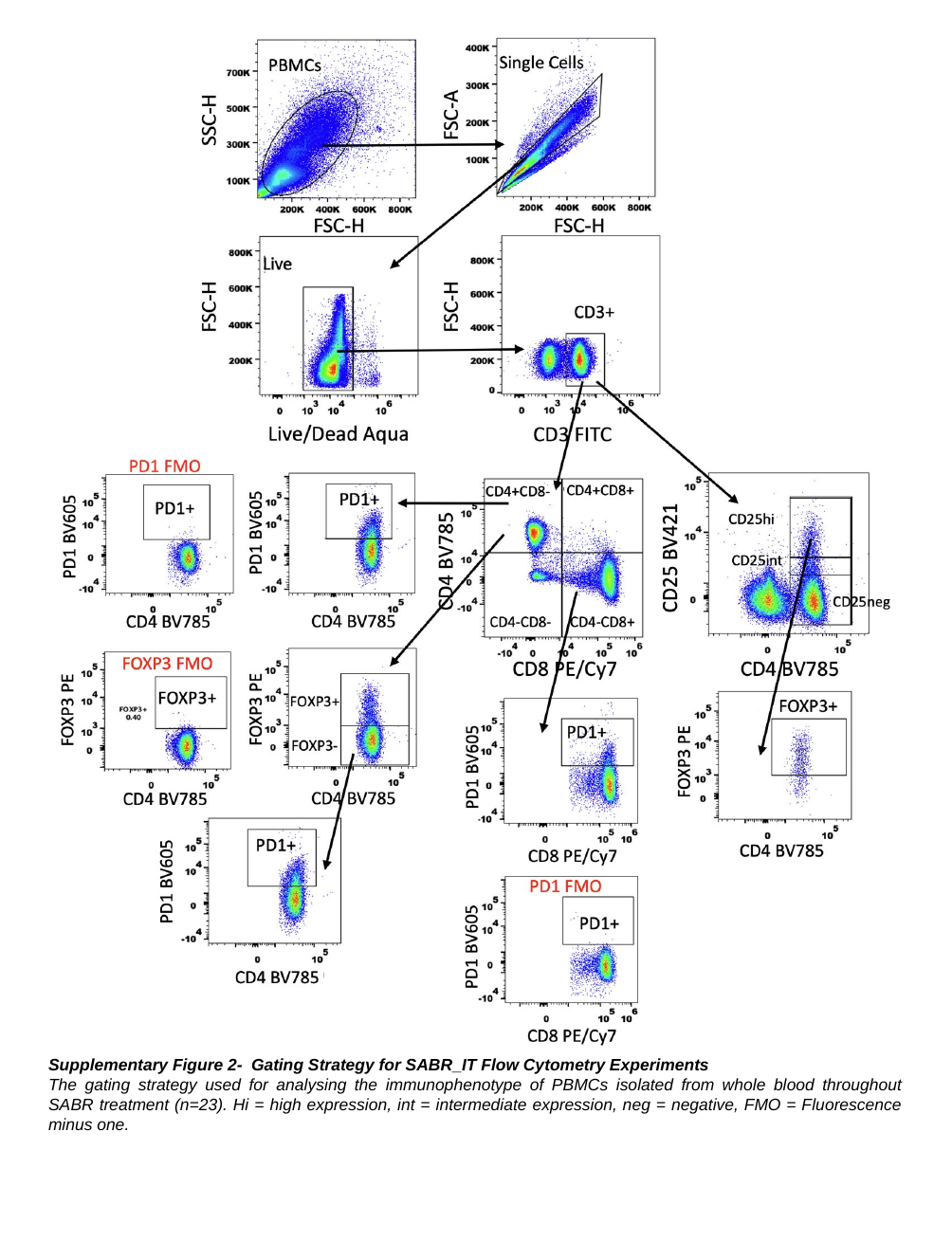

Supplementary Figure 2- Gating Strategy for SABR_IT Flow Cytometry Experiments
The gating strategy used for analysing the immunophenotype of PBMCs isolated from whole blood throughout SABR treatment (n=23). Hi = high expression, int = intermediate expression, neg = negative, FMO = Fluorescence minus one.

## Slide 3
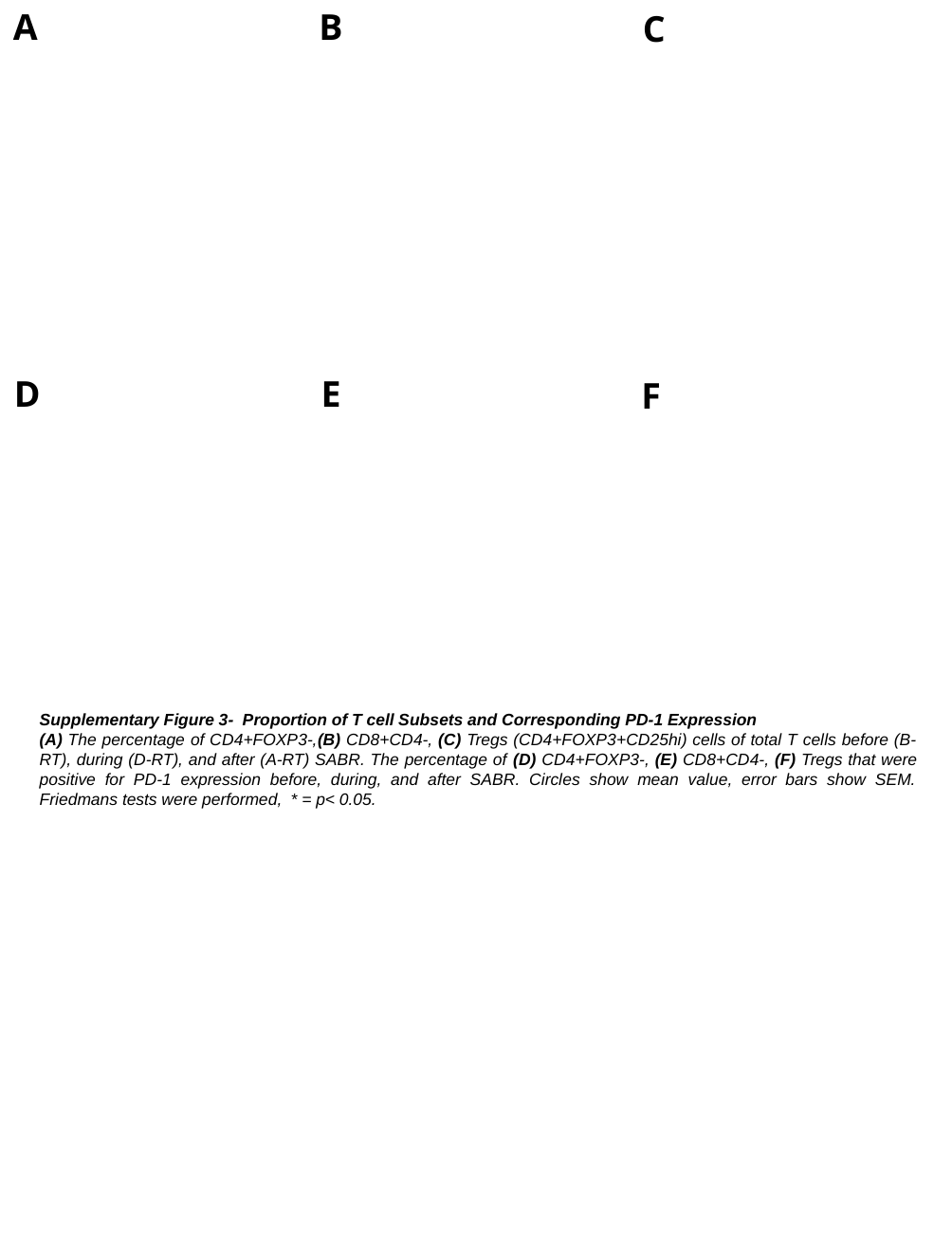

C
A
B
D
E
F
Supplementary Figure 3- Proportion of T cell Subsets and Corresponding PD-1 Expression
(A) The percentage of CD4+FOXP3-,(B) CD8+CD4-, (C) Tregs (CD4+FOXP3+CD25hi) cells of total T cells before (B-RT), during (D-RT), and after (A-RT) SABR. The percentage of (D) CD4+FOXP3-, (E) CD8+CD4-, (F) Tregs that were positive for PD-1 expression before, during, and after SABR. Circles show mean value, error bars show SEM. Friedmans tests were performed, * = p< 0.05.

## Slide 4
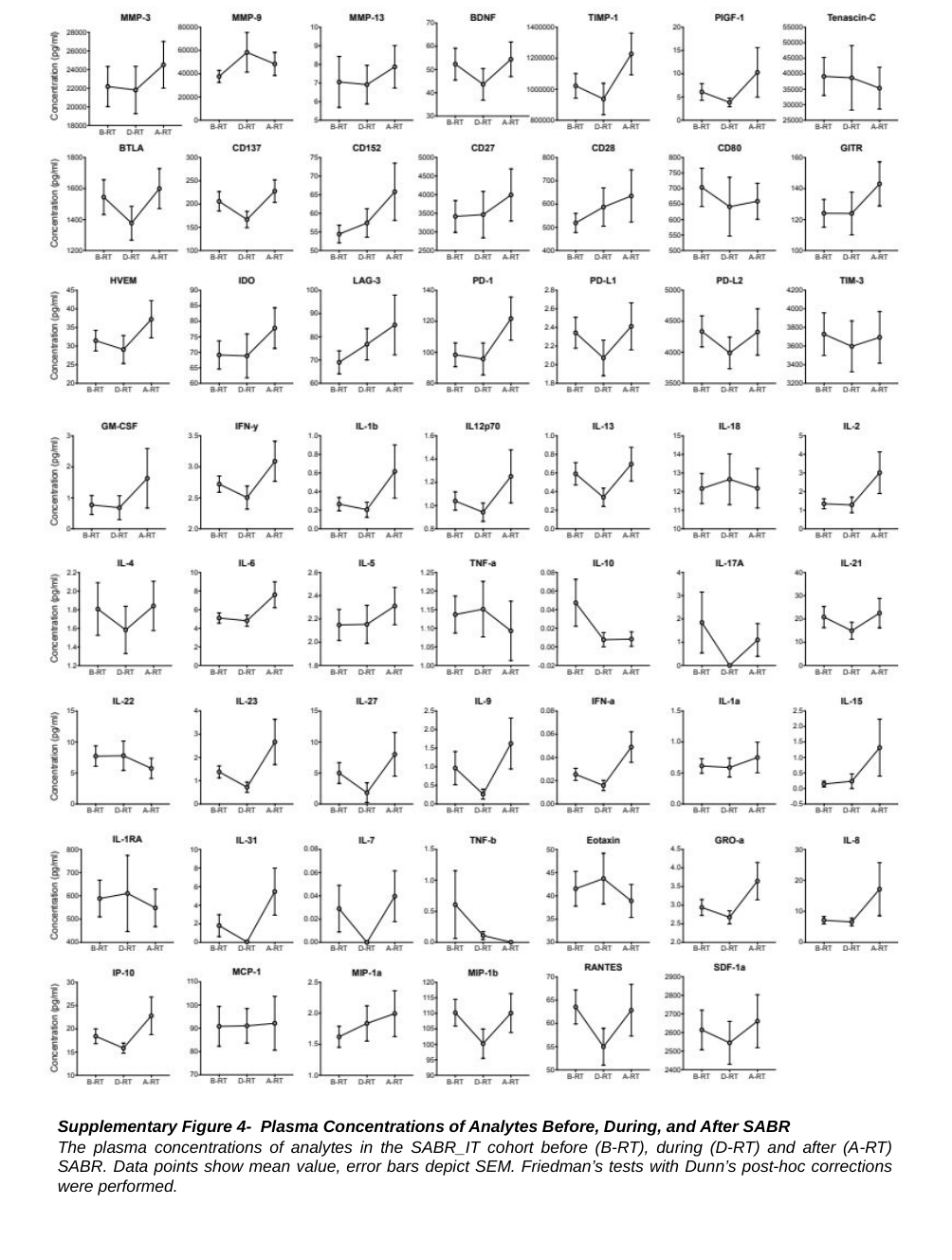

Supplementary Figure 4- Plasma Concentrations of Analytes Before, During, and After SABR
The plasma concentrations of analytes in the SABR_IT cohort before (B-RT), during (D-RT) and after (A-RT) SABR. Data points show mean value, error bars depict SEM. Friedman’s tests with Dunn’s post-hoc corrections were performed.

## Slide 5
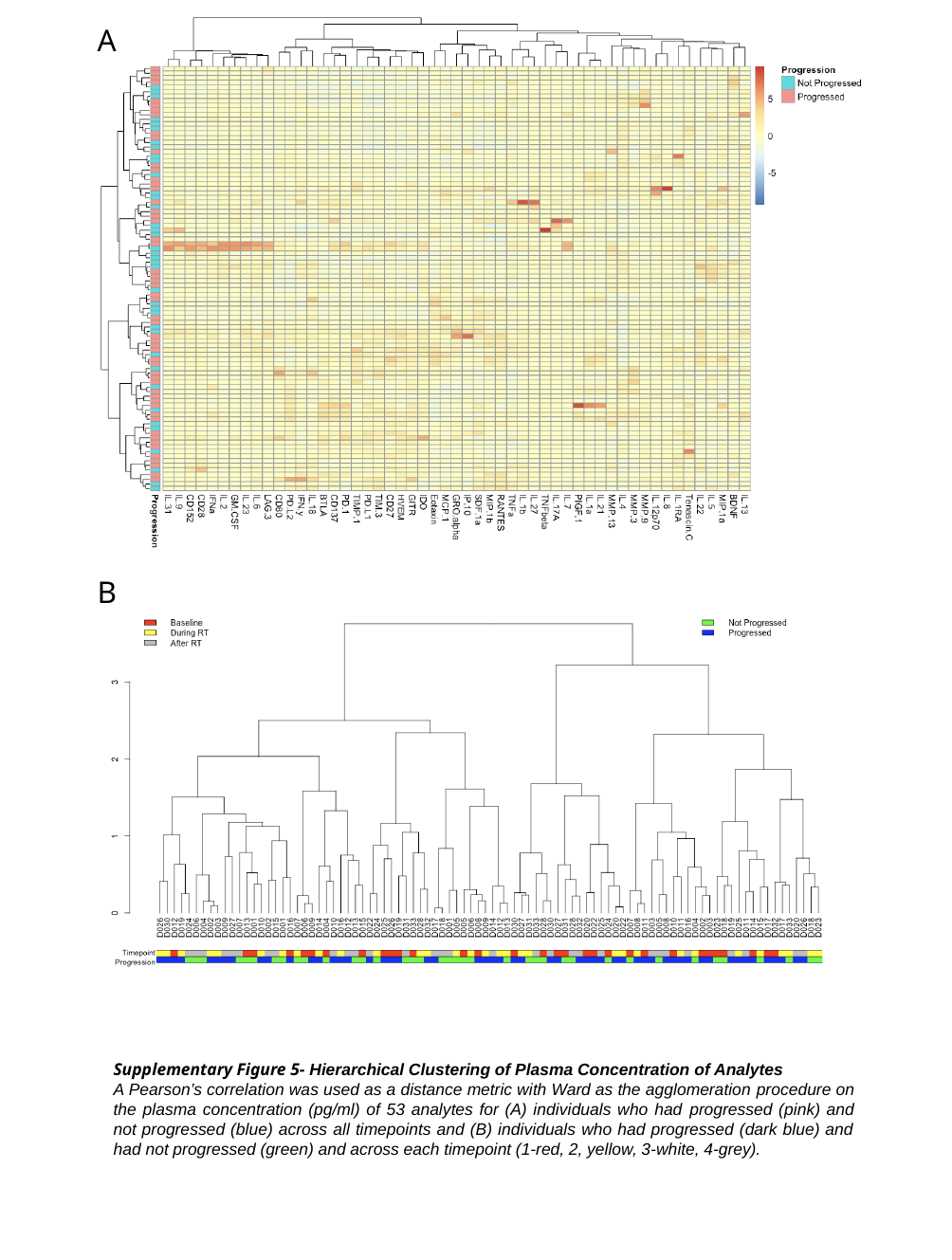

A
B
Supplementary Figure 5- Hierarchical Clustering of Plasma Concentration of Analytes
A Pearson’s correlation was used as a distance metric with Ward as the agglomeration procedure on the plasma concentration (pg/ml) of 53 analytes for (A) individuals who had progressed (pink) and not progressed (blue) across all timepoints and (B) individuals who had progressed (dark blue) and had not progressed (green) and across each timepoint (1-red, 2, yellow, 3-white, 4-grey).

## Slide 6
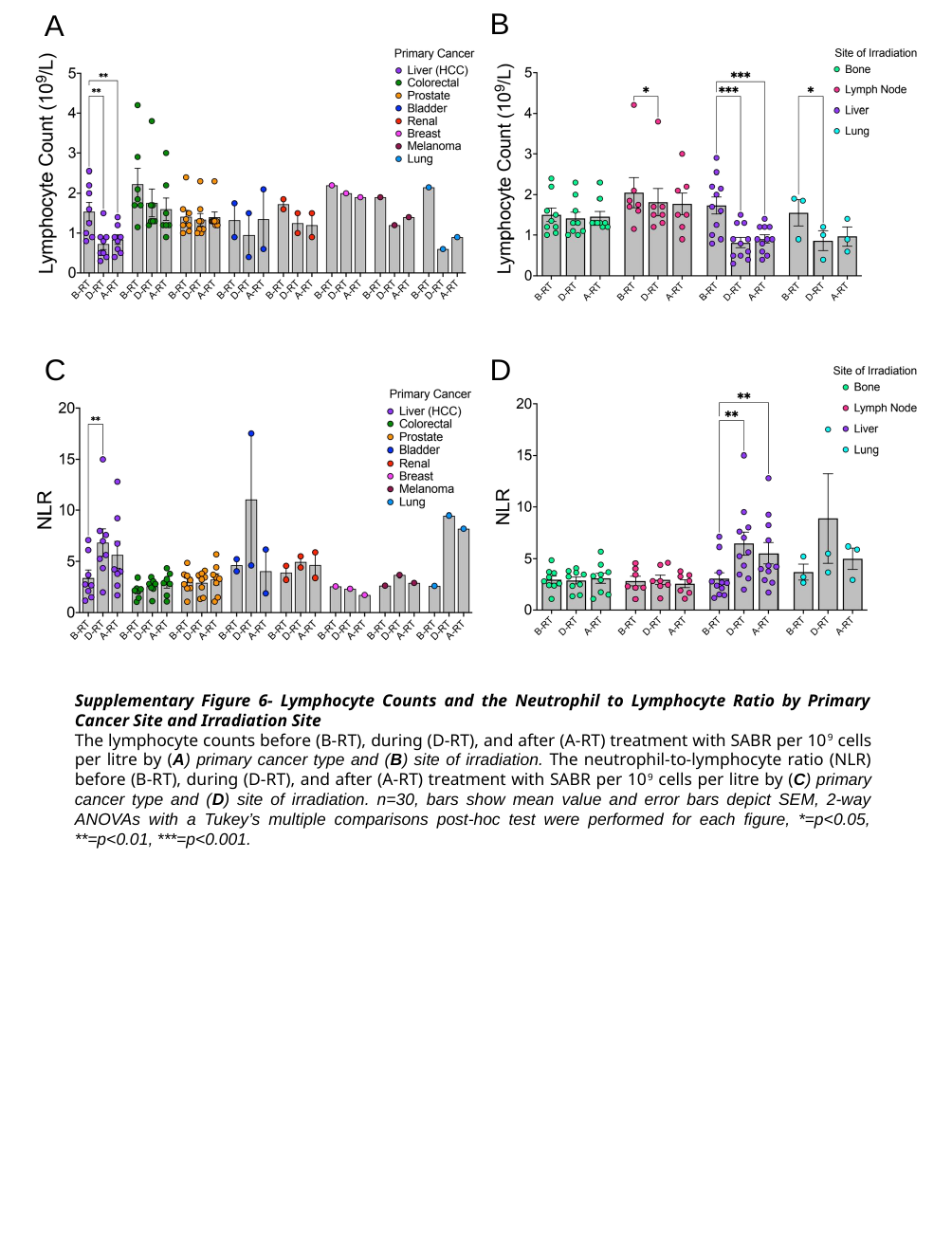

A
B
C
D
Supplementary Figure 6- Lymphocyte Counts and the Neutrophil to Lymphocyte Ratio by Primary Cancer Site and Irradiation Site
The lymphocyte counts before (B-RT), during (D-RT), and after (A-RT) treatment with SABR per 109 cells per litre by (A) primary cancer type and (B) site of irradiation. The neutrophil-to-lymphocyte ratio (NLR) before (B-RT), during (D-RT), and after (A-RT) treatment with SABR per 109 cells per litre by (C) primary cancer type and (D) site of irradiation. n=30, bars show mean value and error bars depict SEM, 2-way ANOVAs with a Tukey’s multiple comparisons post-hoc test were performed for each figure, *=p<0.05, **=p<0.01, ***=p<0.001.

## Slide 7
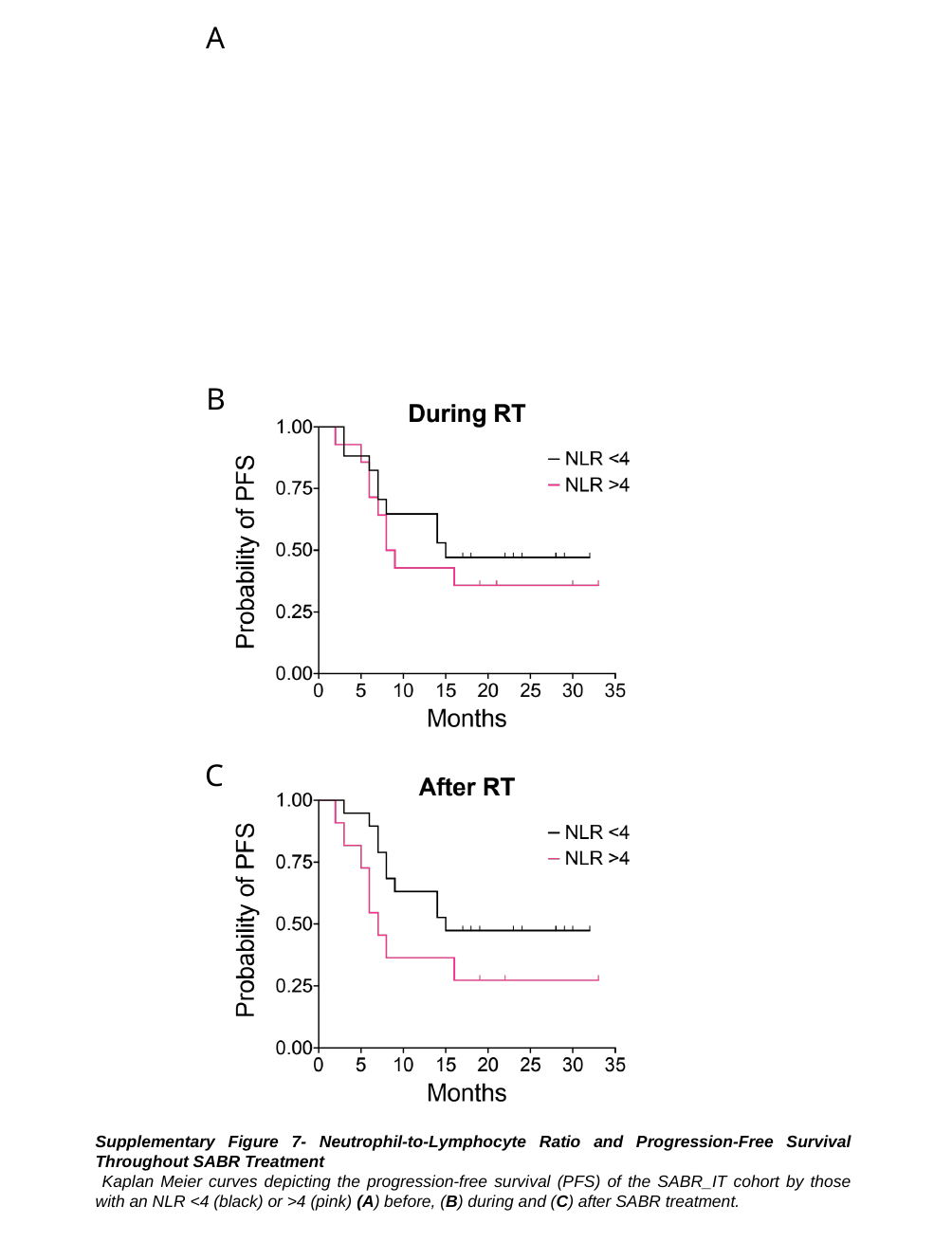

A
B
C
Supplementary Figure 7- Neutrophil-to-Lymphocyte Ratio and Progression-Free Survival Throughout SABR Treatment
 Kaplan Meier curves depicting the progression-free survival (PFS) of the SABR_IT cohort by those with an NLR <4 (black) or >4 (pink) (A) before, (B) during and (C) after SABR treatment.

## Slide 8
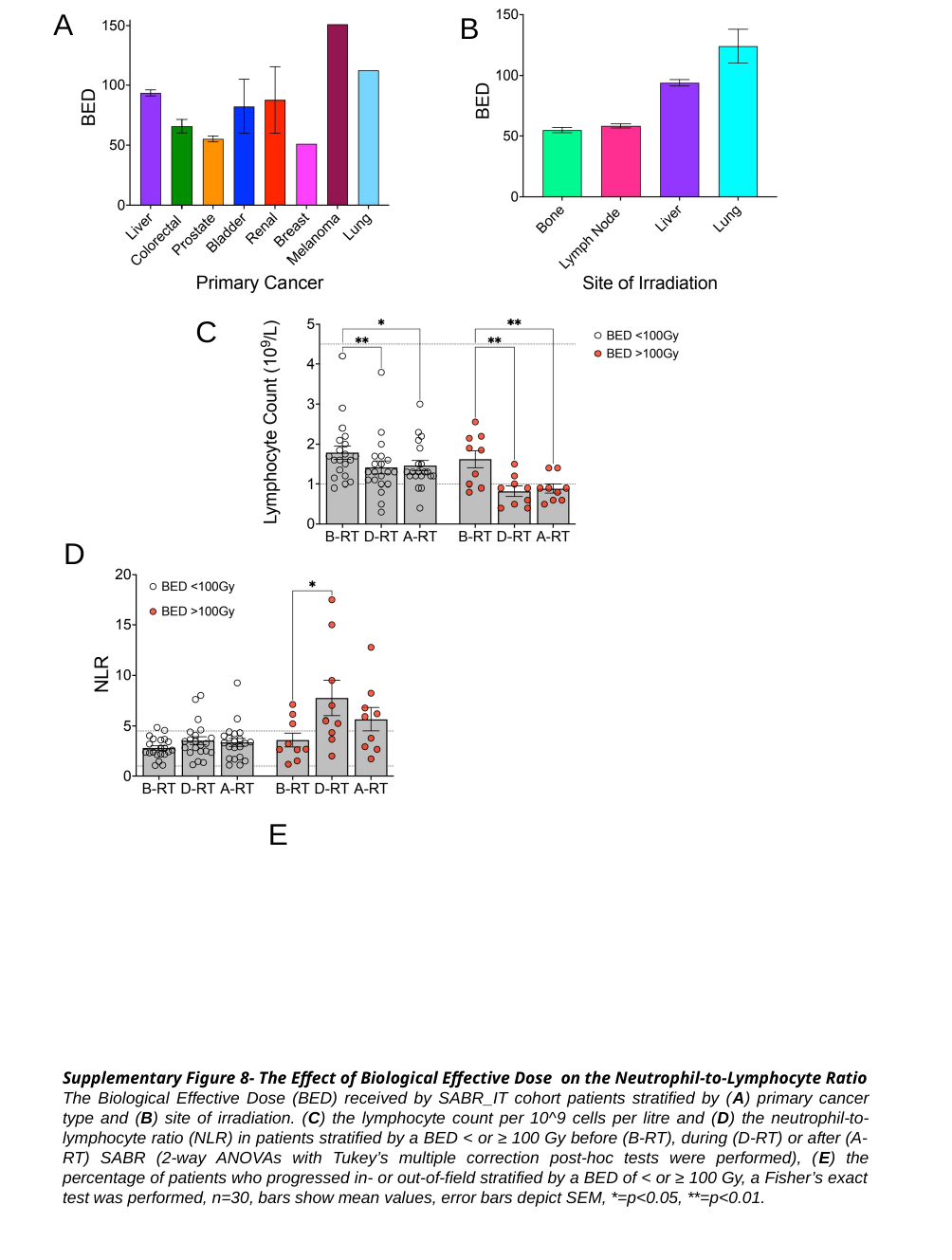

A
B
C
D
E
Supplementary Figure 8- The Effect of Biological Effective Dose on the Neutrophil-to-Lymphocyte Ratio
The Biological Effective Dose (BED) received by SABR_IT cohort patients stratified by (A) primary cancer type and (B) site of irradiation. (C) the lymphocyte count per 10^9 cells per litre and (D) the neutrophil-to-lymphocyte ratio (NLR) in patients stratified by a BED < or ≥ 100 Gy before (B-RT), during (D-RT) or after (A-RT) SABR (2-way ANOVAs with Tukey’s multiple correction post-hoc tests were performed), (E) the percentage of patients who progressed in- or out-of-field stratified by a BED of < or ≥ 100 Gy, a Fisher’s exact test was performed, n=30, bars show mean values, error bars depict SEM, *=p<0.05, **=p<0.01.

## Slide 9
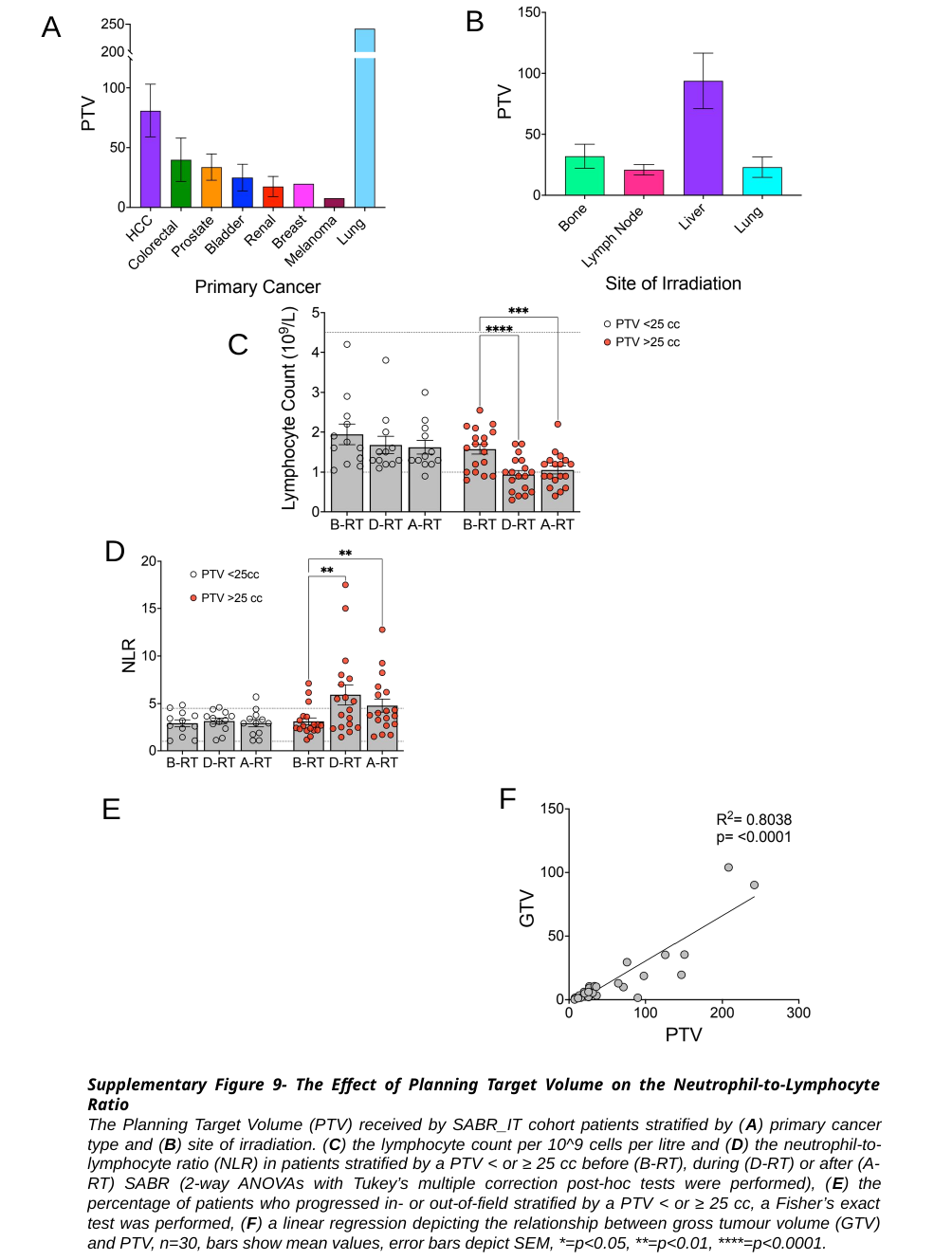

B
A
C
D
F
E
Supplementary Figure 9- The Effect of Planning Target Volume on the Neutrophil-to-Lymphocyte Ratio
The Planning Target Volume (PTV) received by SABR_IT cohort patients stratified by (A) primary cancer type and (B) site of irradiation. (C) the lymphocyte count per 10^9 cells per litre and (D) the neutrophil-to-lymphocyte ratio (NLR) in patients stratified by a PTV < or ≥ 25 cc before (B-RT), during (D-RT) or after (A-RT) SABR (2-way ANOVAs with Tukey’s multiple correction post-hoc tests were performed), (E) the percentage of patients who progressed in- or out-of-field stratified by a PTV < or ≥ 25 cc, a Fisher’s exact test was performed, (F) a linear regression depicting the relationship between gross tumour volume (GTV) and PTV, n=30, bars show mean values, error bars depict SEM, *=p<0.05, **=p<0.01, ****=p<0.0001.

## Slide 10
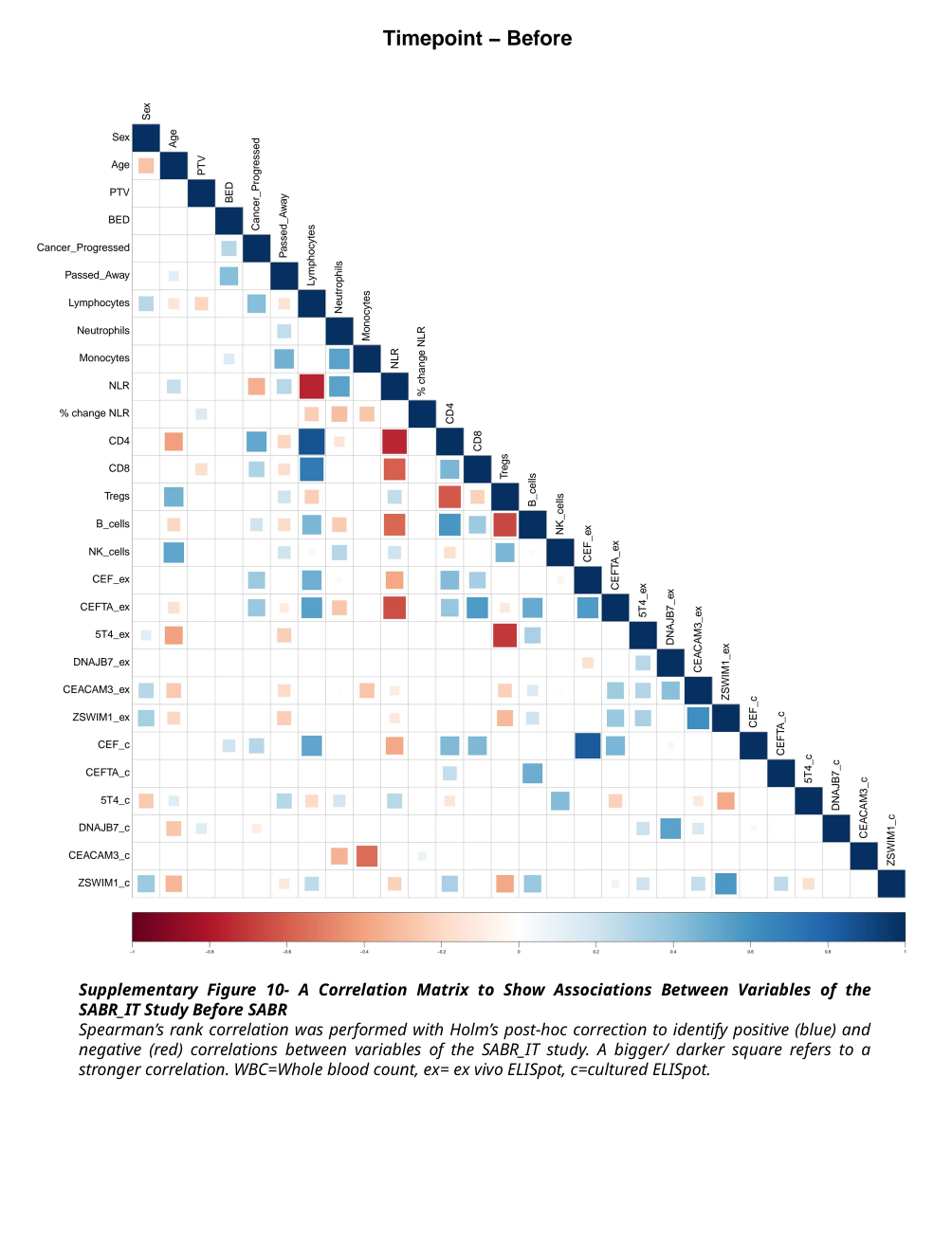

Supplementary Figure 10- A Correlation Matrix to Show Associations Between Variables of the SABR_IT Study Before SABR
Spearman’s rank correlation was performed with Holm’s post-hoc correction to identify positive (blue) and negative (red) correlations between variables of the SABR_IT study. A bigger/ darker square refers to a stronger correlation. WBC=Whole blood count, ex= ex vivo ELISpot, c=cultured ELISpot.

## Slide 11
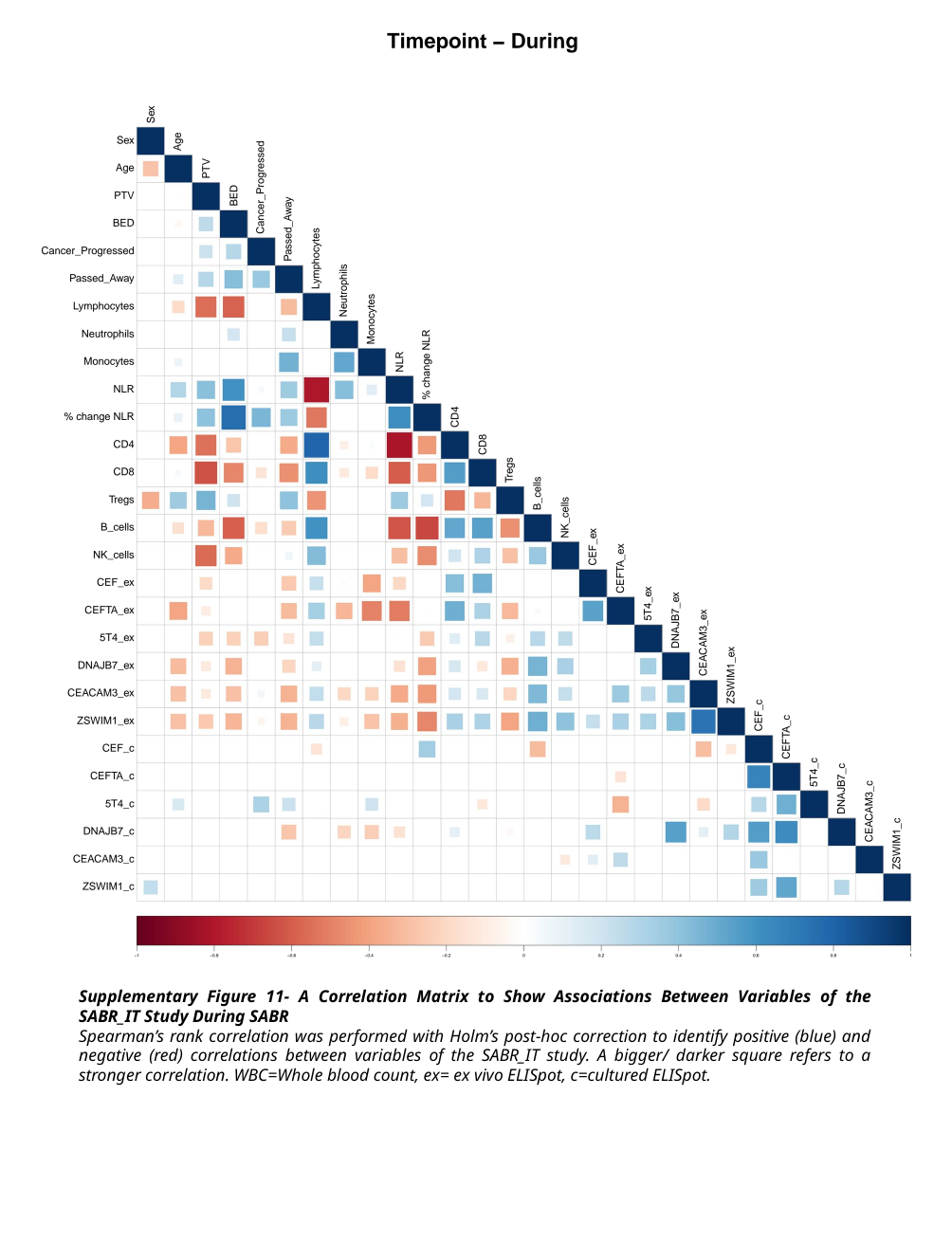

Supplementary Figure 11- A Correlation Matrix to Show Associations Between Variables of the SABR_IT Study During SABR
Spearman’s rank correlation was performed with Holm’s post-hoc correction to identify positive (blue) and negative (red) correlations between variables of the SABR_IT study. A bigger/ darker square refers to a stronger correlation. WBC=Whole blood count, ex= ex vivo ELISpot, c=cultured ELISpot.

## Slide 12
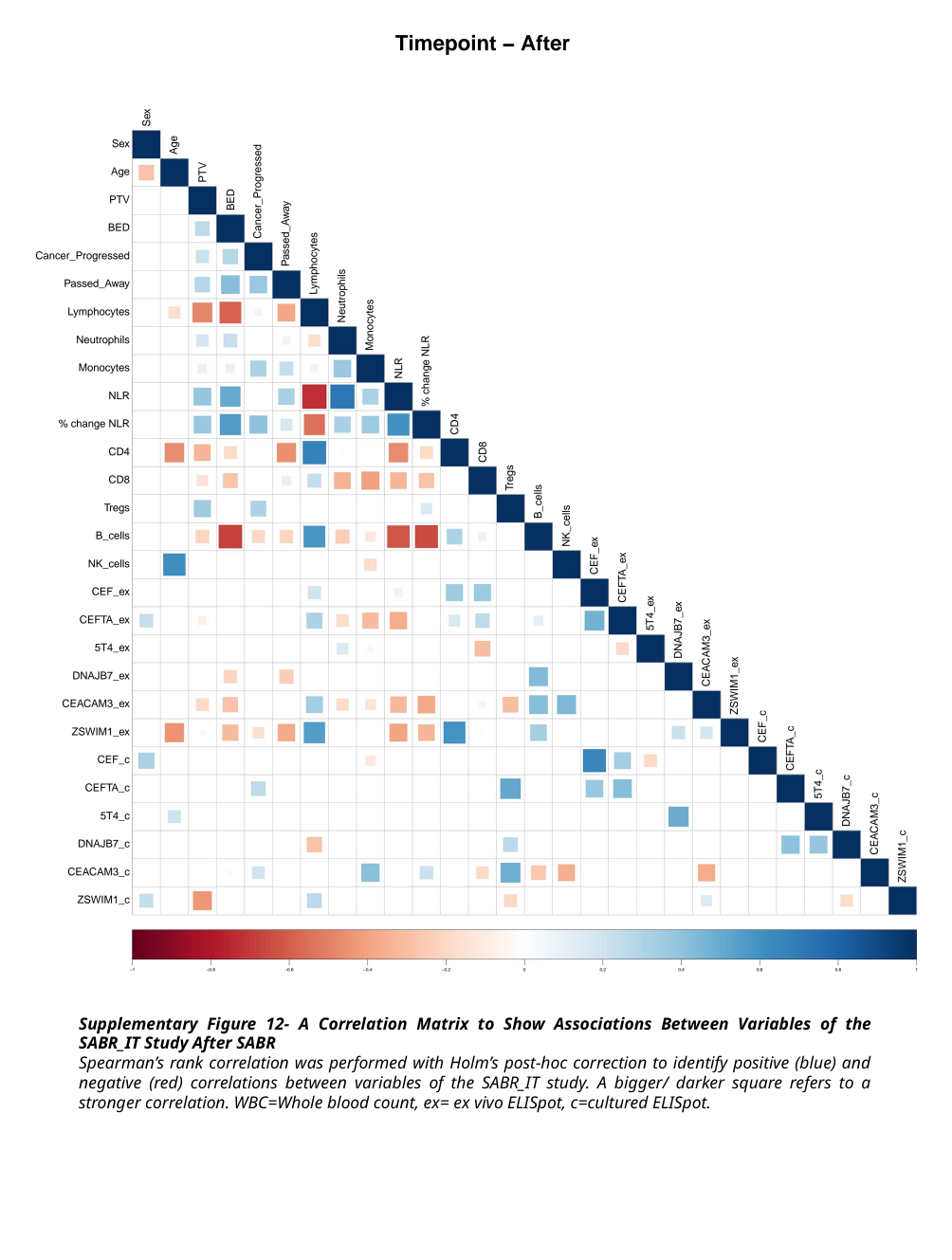

Supplementary Figure 12- A Correlation Matrix to Show Associations Between Variables of the SABR_IT Study After SABR
Spearman’s rank correlation was performed with Holm’s post-hoc correction to identify positive (blue) and negative (red) correlations between variables of the SABR_IT study. A bigger/ darker square refers to a stronger correlation. WBC=Whole blood count, ex= ex vivo ELISpot, c=cultured ELISpot.
