# Supplementary material for: Stereotactic ablative radiotherapy-driven immunosuppression is associated with poorer progression-free survival in cancer patients
Source: Cancer Immunol Immunother. 2025 Dec 18;75(1):3. doi: 10.1007/s00262-025-04218-6 (PMC12715060; doi:10.1007/s00262-025-04218-6)
Supplement: Supplementary file 2 — Supplementary file2 (PPTX 845 KB) [file 262_2025_4218_MOESM2_ESM.pptx]

## Slide 1
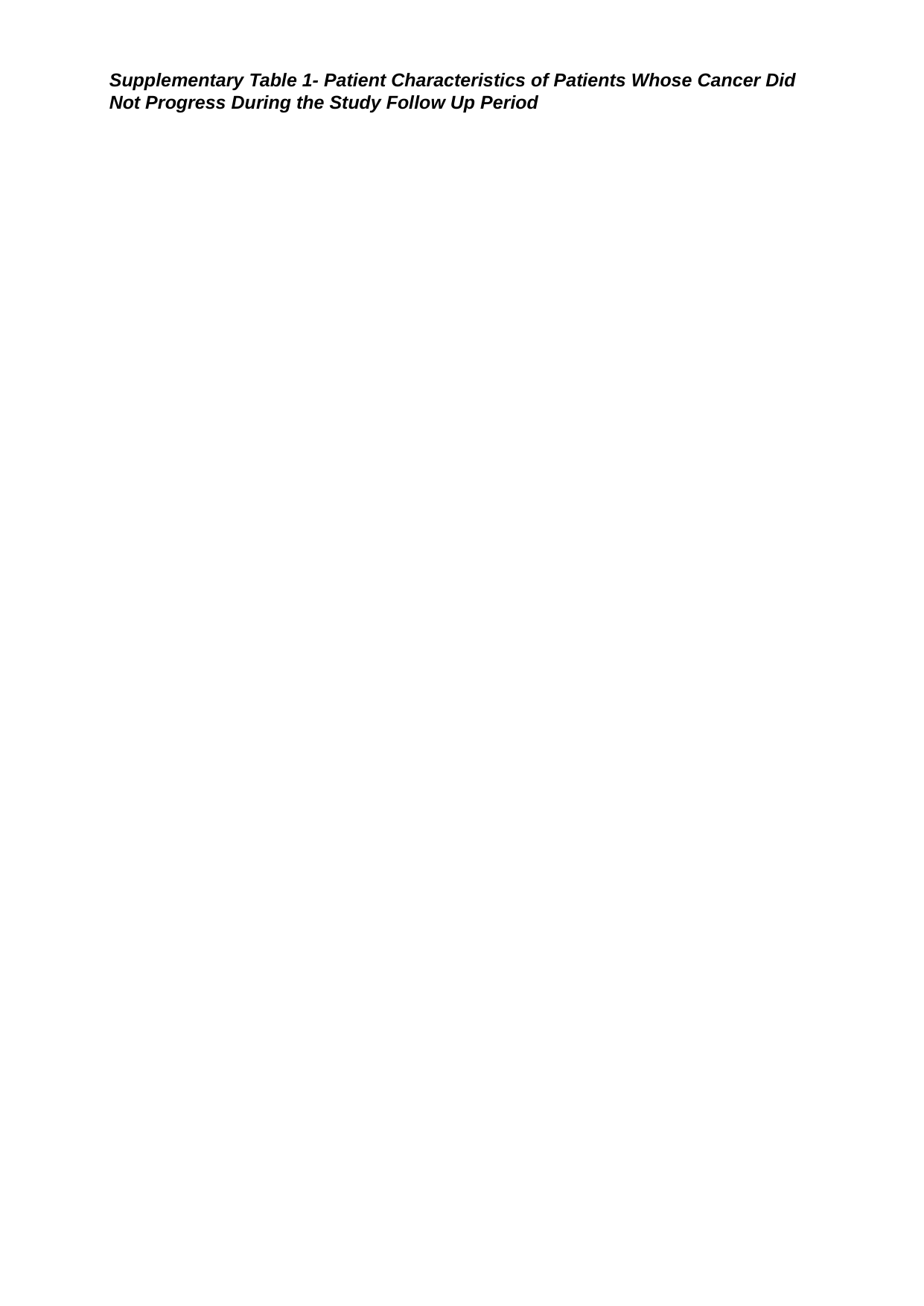

Supplementary Table 1- Patient Characteristics of Patients Whose Cancer Did Not Progress During the Study Follow Up Period

## Slide 2
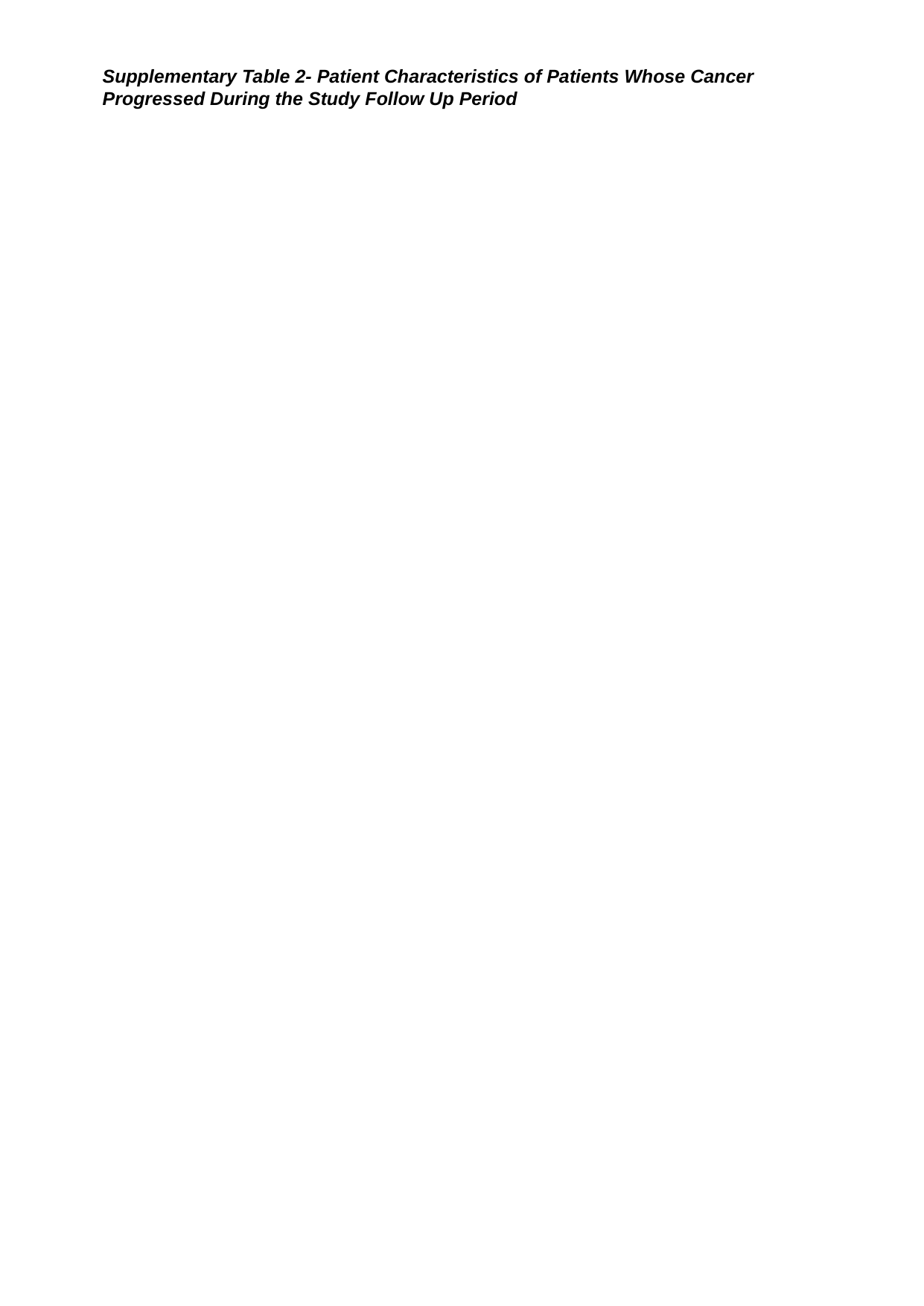

Supplementary Table 2- Patient Characteristics of Patients Whose Cancer Progressed During the Study Follow Up Period

## Slide 3
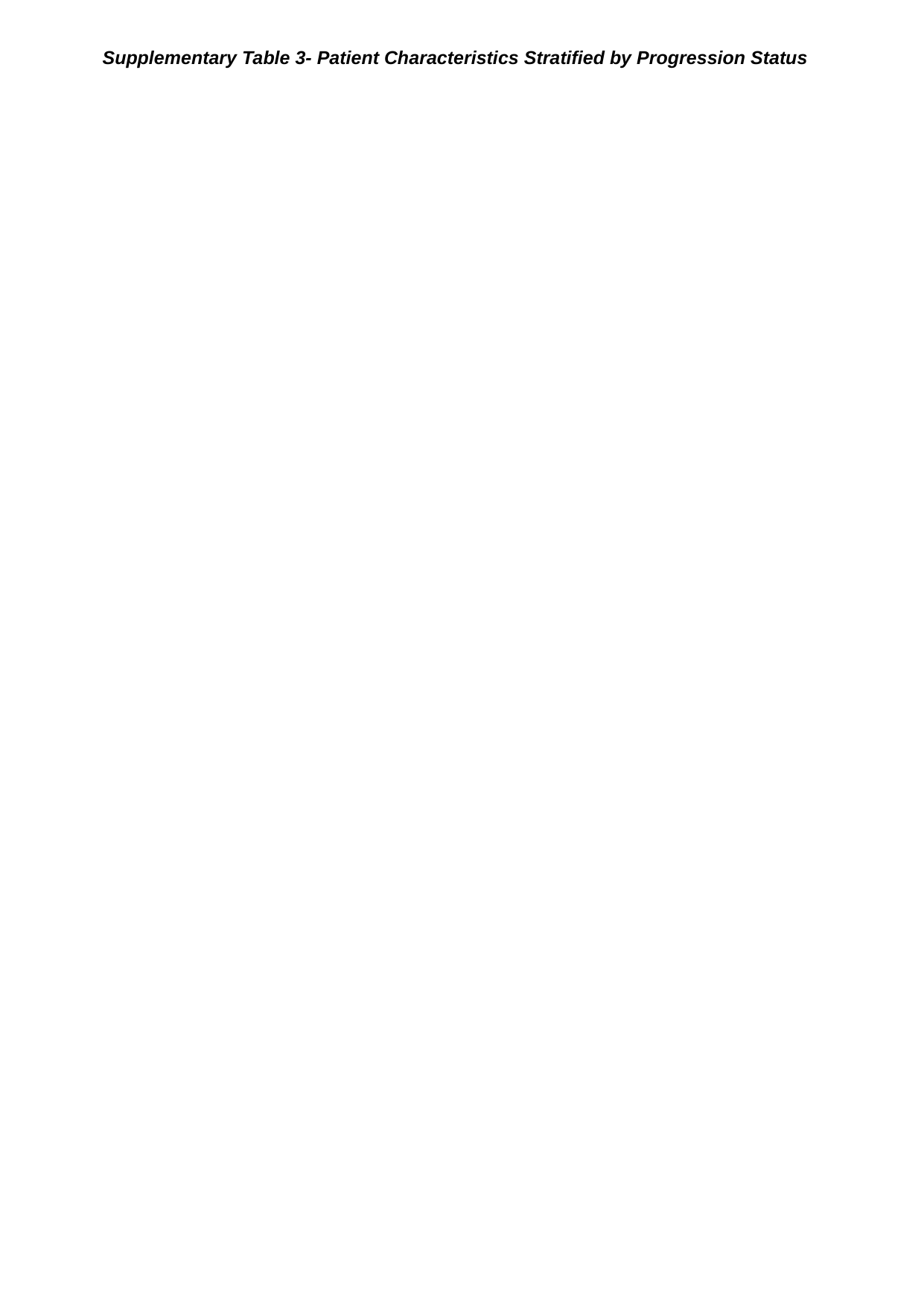

Supplementary Table 3- Patient Characteristics Stratified by Progression Status

## Slide 4
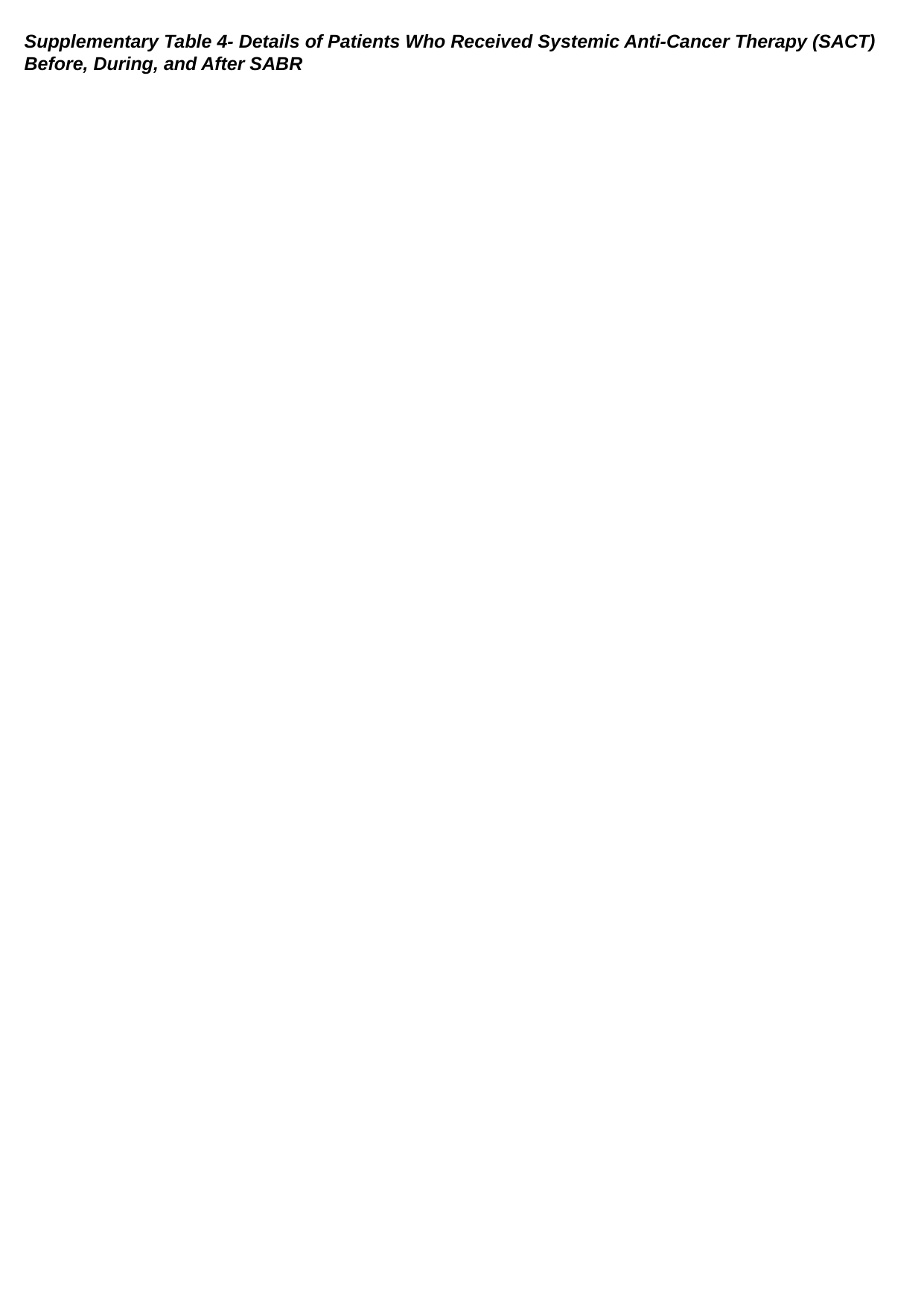

Supplementary Table 4- Details of Patients Who Received Systemic Anti-Cancer Therapy (SACT) Before, During, and After SABR

## Slide 5
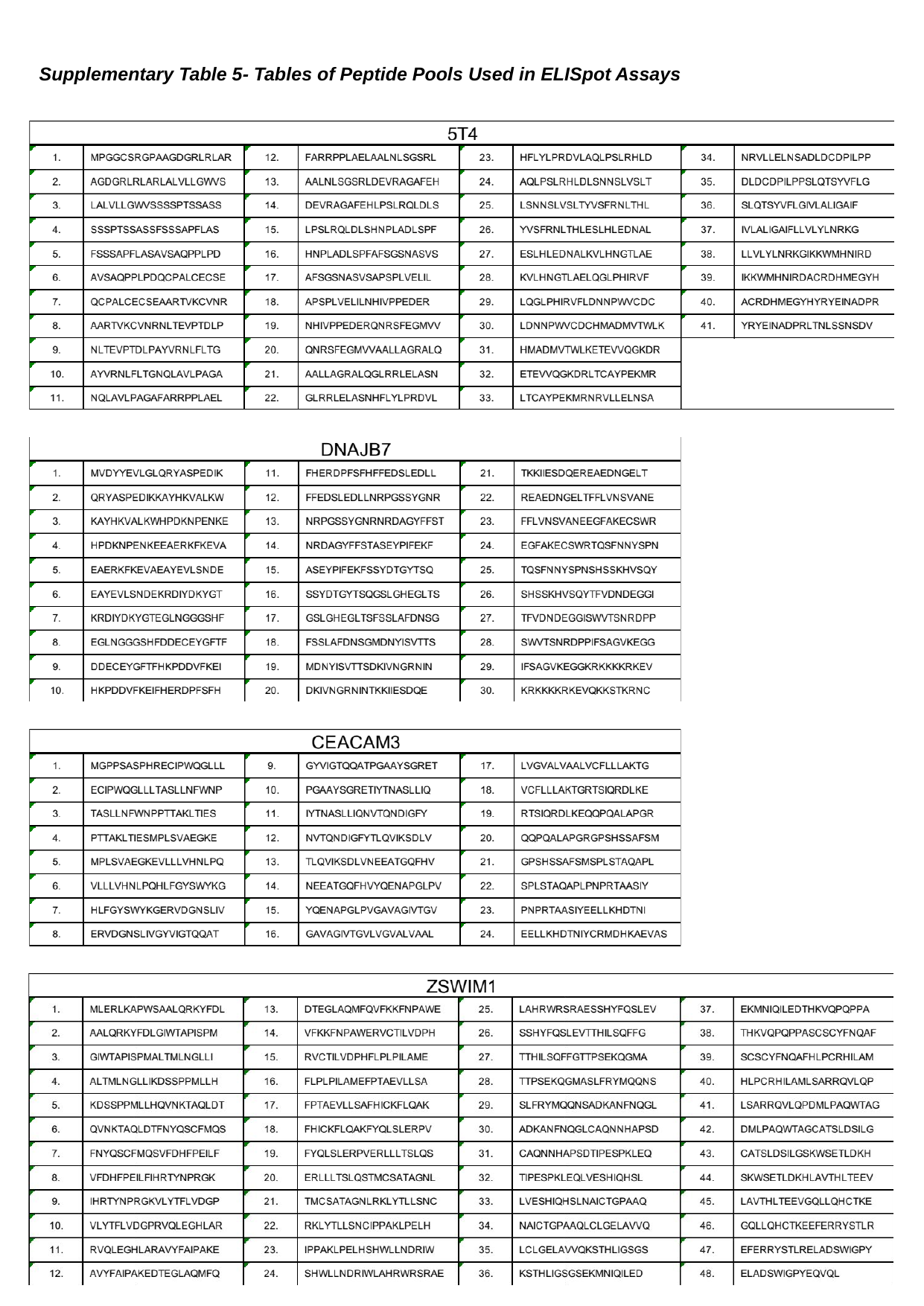

Supplementary Table 5- Tables of Peptide Pools Used in ELISpot Assays

## Slide 6
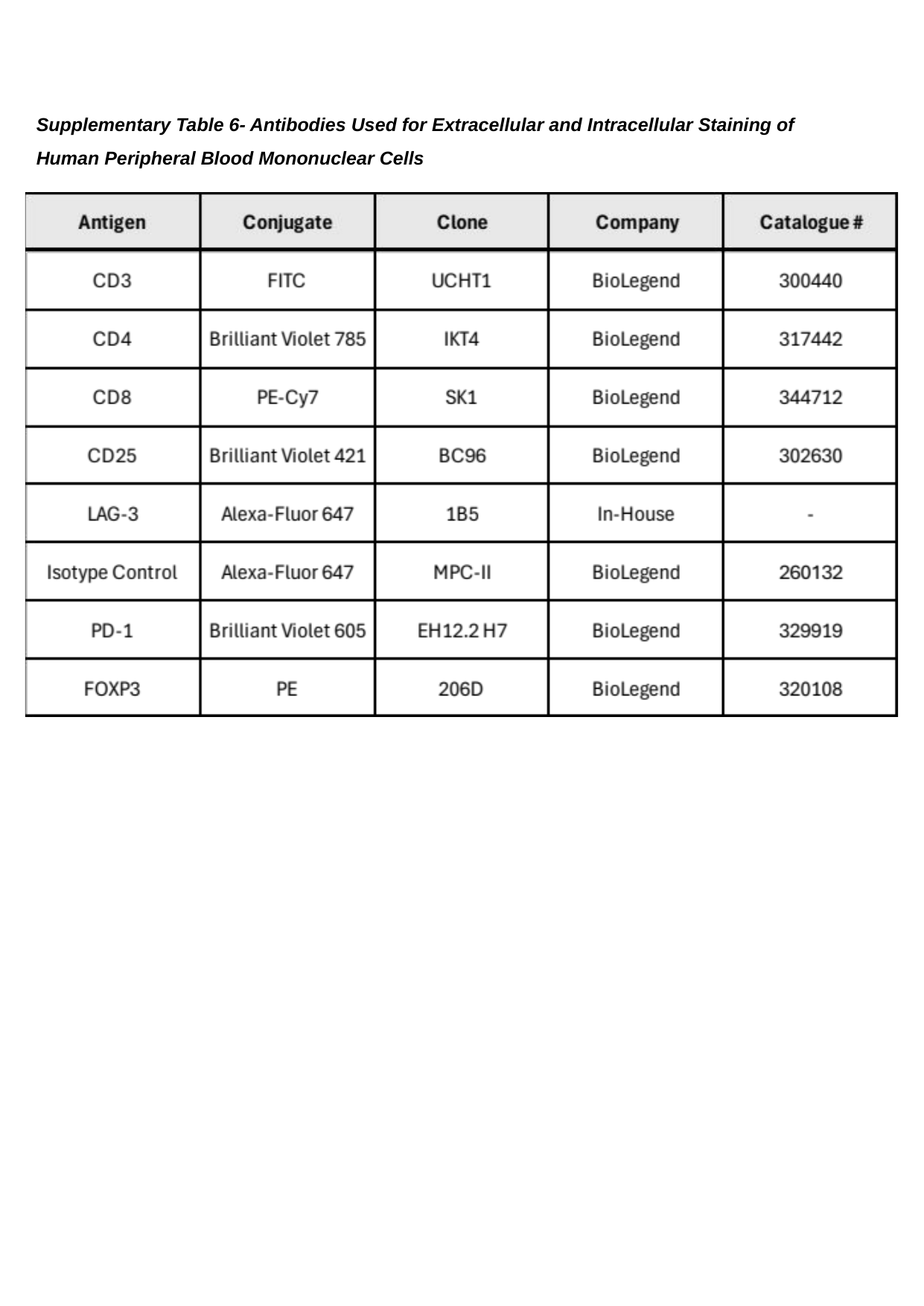

Supplementary Table 6- Antibodies Used for Extracellular and Intracellular Staining of Human Peripheral Blood Mononuclear Cells

## Slide 7
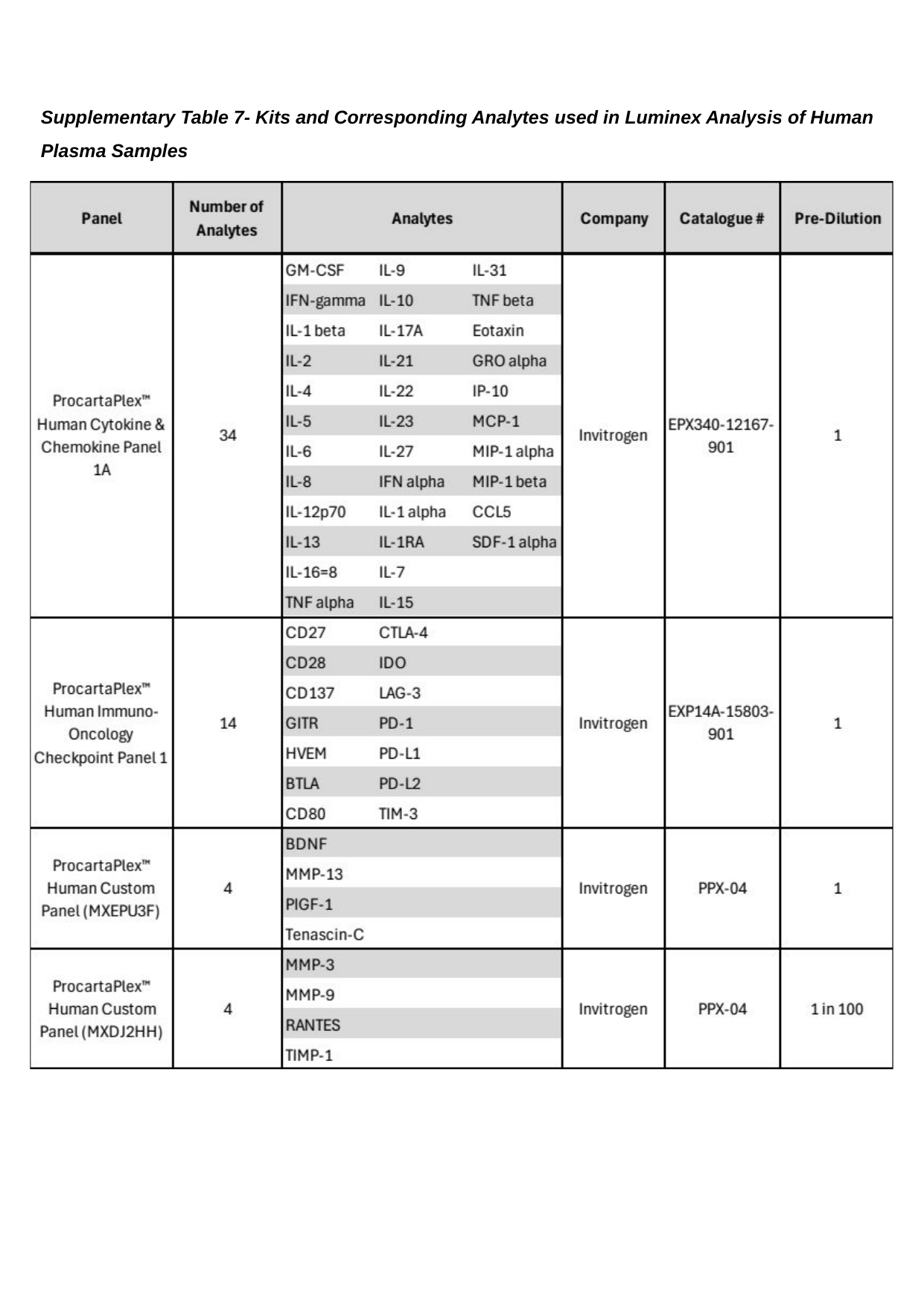

Supplementary Table 7- Kits and Corresponding Analytes used in Luminex Analysis of Human Plasma Samples
